# Aromaticity of Substituted Benzene Derivatives Employing a New Set of Aromaticity Descriptors Based on the Partition of Electron Density

**DOI:** 10.1002/jcc.70257

**Published:** 2025-11-02

**Authors:** Matheus Máximo‐Canadas, Nathália M. P. Rosa, Itamar Borges

**Affiliations:** ^1^ Departamento de Química Rio de Janeiro Rio de Janeiro Brazil

**Keywords:** aromaticity, distributed multipole analysis (DMA), machine learning, ***Q***_2_‐based aromaticity descriptors, substituent effects

## Abstract

Aromatic compounds represent about two‐thirds of known molecules. Despite that, aromaticity still lacks a precise definition, leading to the use of various descriptors to quantify it. In this work, we employ a new set of six descriptors (ACS Omega 2025, **10**, 14157) built from components of the quadrupole moment $Q_{2}$ tensor from the Stone's Distributed Multipole Analysis (DMA) to examine the aromaticity trends in monosubstituted benzene derivatives ($C_{6}H_{5}CH_{3}$, $C_{6}H_{5}CH_{2}^{-}$, $C_{6}H_{5}CH_{2}^{+}$, $C_{6}H_{5}NH_{2}$, $C_{6}H_{5}NH^{-}$, $C_{6}H_{5}NH^{+}$, $C_{6}H_{5}\mathrm{OH}$, $C_{6}H_{5}O^{-}$, and $C_{6}H_{5}O^{+}$) and *para*‐homodisubstituted analogs ($C_{6}H_{4}{\left(CH_{3}\right)}_{2}$, $C_{6}H_{4}{\left(CH_{2}\right)}_{2}$, $C_{6}H_{4}{\left(NH_{2}\right)}_{2}$, $C_{6}H_{4}{\left(\mathrm{NH}\right)}_{2}$, $C_{6}H_{4}{\left(\mathrm{OH}\right)}_{2}$, and $C_{6}H_{4}{\left(O\right)}_{2}$). The $Q_{2}$ tensor is the first term in the DMA electric multipole expansion to include out‐of‐plane electron density contributions. Using a machine learning clustering technique, we identified four groups of molecules: neutral with minimal $Q_{2}$ variation, neutral with strong $Q_{2}$ perturbation ($C_{6}H_{4}{\left(O\right)}_{2}$ and $C_{6}H_{4}{\left(NH_{2}\right)}_{2}$), positively, and negatively charged species. Comparisons with the traditional aromaticity descriptors HOMA, $\mathrm{AI}\left(\mathrm{vib}\right)$, $I_{\text{ring}}$, NICS, $\mathrm{EL}F_{\pi }$, and MCI were carried out. Among the $Q_{2}$‐based descriptors, $Q_{2}{\left(1\right)}_{\mathrm{zz}}$, which is computed 1 Å above the ring plane, exhibited the highest correlation with those descriptors, though small ($R^{2}\approx 0.6$). All the $Q_{2}$‐based descriptors reproduced the same trends of the traditional indices, except for the negative substituents, which were only correctly described by the former ones. We found that $Q_{2}$‐based descriptors correlated with ionization potentials (IPs), electron affinities (EAs), electronegativities (χ), electrophilicity indexes (ω), and electron‐donating/accepting power ($\omega^{-}$, $\omega^{+}$), but not with the chemical hardness (η). In contrast, $I_{\text{ring}}$, NICS, $\mathrm{EL}F_{\pi }$, and MCI correlated only with η, while HOMA and $\mathrm{AI}\left(\mathrm{vib}\right)$ showed no correlation. Therefore, our results show that the $Q_{2}$‐based descriptors reflect the π‐electron delocalization characteristic of aromaticity and also can describe frontier‐orbital reactivity trends.

## Graphical abstract

We applied to substituted benzene derivatives a new set of aromaticity descriptors, based on the distributed multipole analysis (DMA) components of the quadrupole moment $Q_{2}$ tensor, the first one in the DMA expansion including contributions from the out‐of‐plane electron distribution. The $Q_{2}$‐based descriptors were used to examine their aromaticity, and compared with traditional indices. The results showed that the new indices could describe the main trends of the traditional ones, except for the negative substituents.

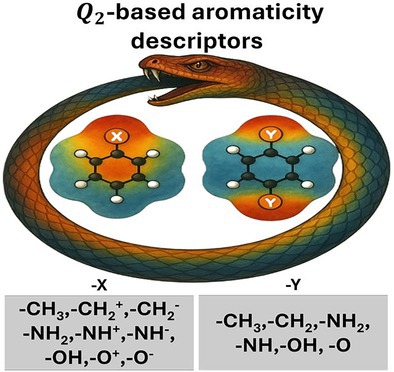

## 1 Introduction

Aromaticity is a theoretical concept developed to rationalize a series of experimental observations displayed by a particular class of compounds [1]. These include bond length equalization, exceptional thermodynamic stability, distinctive magnetic behavior, such as the emergence of induced ring currents, and a characteristic reactivity that favors substitution reactions over addition. Therefore, aromaticity serves as a unifying conceptual tool that brings coherence to what would otherwise be incongruent experimental findings [2].

Benzene was first isolated by Michael Faraday in 1825, drawing attention for its unusual stability among unsaturated hydrocarbons [3]. In 1865, the German chemist August Kekulé proposed a hexagonal structure with alternating double bonds, later suggesting that their dynamic delocalization explains this stability [4]. The German physicist Erich Hückel's 1931 formulation of the 4n+2 rule for the π‐electron count became a foundational criterion for aromaticity [5, 6]. However, not all compounds following this rule exhibit aromatic behavior. Since the Jerusalem Conference in 1960, a consensus has arisen that aromatic compounds must be planar, cyclic, and contain a delocalized system of conjugated electrons [7]. While traditionally associated with organic systems, aromaticity has since been extended to a broad range of nonconventional species [8]. These systems, while sharing some characteristics with classical aromatics, also display distinct properties, prompting the introduction of various subcategories such as Möbius aromaticity [9], homoaromaticity [10], heteroaromaticity [11], Clar aromaticity [12], three‐dimensional aromaticity [13], metalloaromaticity [14], and *σ*‐aromaticity [15, 16], among others [17].

Given the vast diversity of aromatic systems, efforts have been made to establish quantitative criteria for aromaticity. However, although two‐thirds of known compounds are classified as aromatic, there is no consensus on what aromaticity is [18]. Aromaticity is not a directly measurable quantity because it is not observable in the quantum mechanical sense. In other words, there is no quantum operator corresponding to a measurable aromaticity [19]. This epistemological challenge is not unique to aromaticity. Still, it extends to numerous foundational concepts in chemistry, including electronegativity, van der Waals radii, resonance, orbitals, chemical bonding, atomic charges, oxidation states, local spin, and bond order, among others [2, 20]. Moreover, these concepts remain indispensable in chemistry, not due to their physical concreteness, but because of their heuristic power in explaining and predicting molecular behavior [21].

Given its importance and because aromaticity cannot be measured directly, various aromaticity descriptors (or indices) have been developed to assess properties commonly associated with aromatic systems. These descriptors are theoretical parameters designed to quantify and rationalize the degree of aromatic character in chemical compounds [22]. As a theoretical construct, however, the meaning of aromaticity is inherently model‐dependent [23]. Therefore, the classification of aromatic or non‐aromatic molecules depends on the definition of aromaticity [24]. This has led to the development of various descriptors to quantify the degree of aromatic character indirectly [25]. These descriptors can be based on electron delocalization [26], energetic stabilization [27], bond length equalization [28], and magnetic behavior [29].

Among the magnetic‐based aromaticity descriptors, the *Nucleus‐Independent Chemical Shift* (NICS) is among the most widely employed [30]. It estimates the presence of induced electronic ring currents in aromatic systems by calculating the magnetic shielding at the center or above the plane of the ring [31]. From a structural stance, the *Harmonic Oscillator Model of Aromaticity* (HOMA) is commonly used to quantify aromaticity based on the uniformity of bond lengths within the ring [32]. In the thermodynamic domain, the *Isomerization Stabilization Energy* (ISE) assesses the energetic stabilization associated with forming an aromatic system [33]. In the vibrational domain, the *Aromaticity Index from Vibrational Data* ($\mathrm{AI}\left(\mathrm{vib}\right)$) uses local vibrational force constants to estimate the degree of aromaticity [34]. The *Electron Localization Function* (ELF) is a topological descriptor derived from the electronic density, which enables the analysis of electron pair localization in a molecule [35]. ELF has also been partitioned into its *σ* and *π* components, denoted as $\mathrm{EL}F_{\sigma }$ and $\mathrm{EL}F_{\pi }$, respectively [36, 37]. In the electronic domain, descriptors such as the *Fluctuation Index* (FLU) and the *Multicenter Index* (MCI) are particularly used as they evaluate π‐electron delocalization and multi‐center electron correlation within conjugated systems [38, 39]. Since there is no universal descriptor, each of these descriptors offers a complementary perspective on the multifaceted phenomenon of aromaticity and is typically selected according to the specific purpose of the study [40]. An excellent and up‐to‐date analysis and presentation of aromaticity and its various descriptors can be found in the book by Solà and coauthors [41].

According to Lazzeretti [40], aromaticity descriptors require caution, as they all present conceptual and methodological limitations. Structural descriptors, for instance, may reflect factors such as angle strain or steric effects, which are not necessarily related to electron delocalization. Energetic descriptors rely on arbitrarily chosen reference systems and are susceptible to interference from various structural effects, making it difficult to isolate the aromatic contribution. Magnetic descriptors, such as NICS, also risk misinterpretation, as they can be influenced by local shielding or deshielding effects, which do not necessarily correspond to the presence of characteristic aromatic ring currents. Moreover, NICS cannot distinguish between local currents (e.g., those associated with lone pairs) and global ring currents.

While it can be an oversimplification to reduce aromaticity solely to electron delocalization due to the out‐of‐plane π‐electrons, this aspect remains the most critical factor in determining the aromatic character [8, 26]. In this context, our research group has recently developed a new set of six aromaticity descriptors based on the electron density delocalization using Stone's Distributed Multipole Analysis (DMA) method for partitioning the molecular electron density [42, 43]. DMA partitions the electron density into atomic‐centered electric multipoles localized at different points of the molecule [44, 45]. This new set of descriptors is based on the components of the quadrupole moment $Q_{2}$ tensor, the first in the DMA expansion to include out‐of‐plane electron components. These descriptors have shown promising performance across the 12 test sets of aromatic and antiaromatic molecules from the Girona benchmark proposed by the Solà group [8, 46]. We have recently shown that this approach successfully captures the impact of fluorination on the aromaticity of 12 benzene‐based compounds [47].

In this work, we apply our DMA $Q_{2}$‐based descriptors [48] to examine the effect of substituents on the benzene ring's aromatic character, previously studied with other descriptors [49]. Electron‐donating and electron‐withdrawing substituents can significantly modulate the electron density of the aromatic ring [50]. This modification of the electron distribution can influence the resonance patterns and π‐electron delocalization, two key factors that affect aromaticity [50, 51]. We aimed to evaluate the sensitivity and robustness of the descriptors in capturing subtle electronic modifications induced by different electron‐donating and electron‐withdrawing groups.

## 2 Theoretical Methods

**FIGURE 1:**
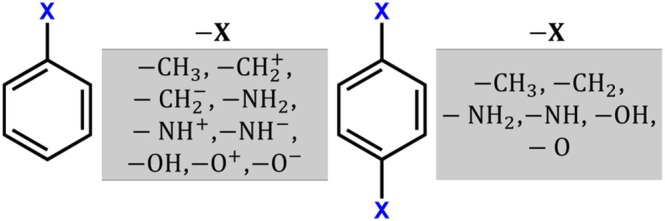
The chemical structures of the investigated molecules.

To enable a robust comparison with other descriptors reported in the literature, the optimized geometries of monosubstituted benzene derivatives ($C_{6}H_{5}CH_{3}$, $C_{6}H_{5}CH_{2}^{-}$, $C_{6}H_{5}CH_{2}^{+}$, $C_{6}H_{5}NH_{2}$, $C_{6}H_{5}NH^{-}$, $C_{6}H_{5}NH^{+},$ $C_{6}H_{5}\mathrm{OH},$ $C_{6}H_{5}O^{-},$ and $C_{6}H_{5}O^{+}$) and *para*‐homodisubstituted analogs ($C_{6}H_{4}{\left(CH_{3}\right)}_{2}$, $C_{6}H_{4}{\left(CH_{2}\right)}_{2}$, $C_{6}H_{4}{\left(NH_{2}\right)}_{2}$, $C_{6}H_{4}{\left(\mathrm{NH}\right)}_{2}$, $C_{6}H_{4}{\left(\mathrm{OH}\right)}_{2}$, and $C_{6}H_{4}{\left(O\right)}_{2}$) were obtained from the work of Chagas and collaborators who computed several traditional aromaticity descriptors, namely HOMA, $\mathrm{AI}\left(\mathrm{vib}\right)$, BVI, MCI, $\text{NICS}\left(0\right)$, $\text{NICS}\left(1\right)$, and $\text{NICS}{\left(1\right)}_{\mathrm{zz}}$ [49]. The chemical structures are presented in Figure 1, and the xyz coordinates of the converged geometries are collected in Table S4. They optimized the geometries using the DFT hybrid exchange‐correlation functional B3LYP [52, 53] and the def2‐TZVP basis set [54, 55], employing an ultrafine integration grid and tight convergence thresholds ($10^{-8}$ for SCF energy and $10^{-8}$ atomic units for geometry optimizations).

Several studies have examined the relationship between aromatic character and substituent effects [47, 50, 51, 56, 57]. However, Krygowski and co‐workers showed that the influence of substituents on π‐electron delocalization in *meta*‐ and *para*‐disubstituted benzene derivatives is comparable for many substituents [50, 56]. Therefore, the dataset selected from Chagas et al. [49], which comprises strong donor and acceptor groups as well as neutral, anionic, and cationic species, covers a representative range of electron density variations in the benzene core. Furthermore, given that this dataset was used to compute several aromaticity descriptors (HOMA, $\mathrm{AI}\left(\mathrm{vib}\right)$, NICS, among others), it enables a direct comparison between our new $Q_{2}$‐based descriptors and widely used traditional indices. It should also be emphasized that the primary purpose of the present study is to evaluate the sensitivity and robustness of the new descriptors against a representative and previously investigated dataset.

We then carried out single‐point energy calculations to obtain the necessary electron densities for computing the $Q_{2}$‐based aromaticity descriptors at the same theoretical level of the converged geometries (B3LYP/def2‐TZVPD). The Gaussian 16 Revision A03 was used for these calculations. As detailed in our previous work, where we proposed the new aromaticity descriptors [48], two keywords were employed in the Gaussian input file: *nosymm* and *Density = Current*. The first ensures that no symmetry‐based reorientation of the molecular frame occurs during the single‐point electron density calculation. This secures that the origin of the coordinate system remains centered at the geometric center of the ring and that the ring remains on the *xy*‐plane. The *Density = Current* keyword instructs Gaussian to use the updated electron density from the converged SCF calculation. This guarantees that all calculated properties are based on the electron density that truly corresponds to the optimized geometry. For a detailed definition of the $Q_{2}$‐based aromaticity descriptors and their mathematical formulation, the reader is referred to Section 3 of the Supporting Information.

**FIGURE 2:**
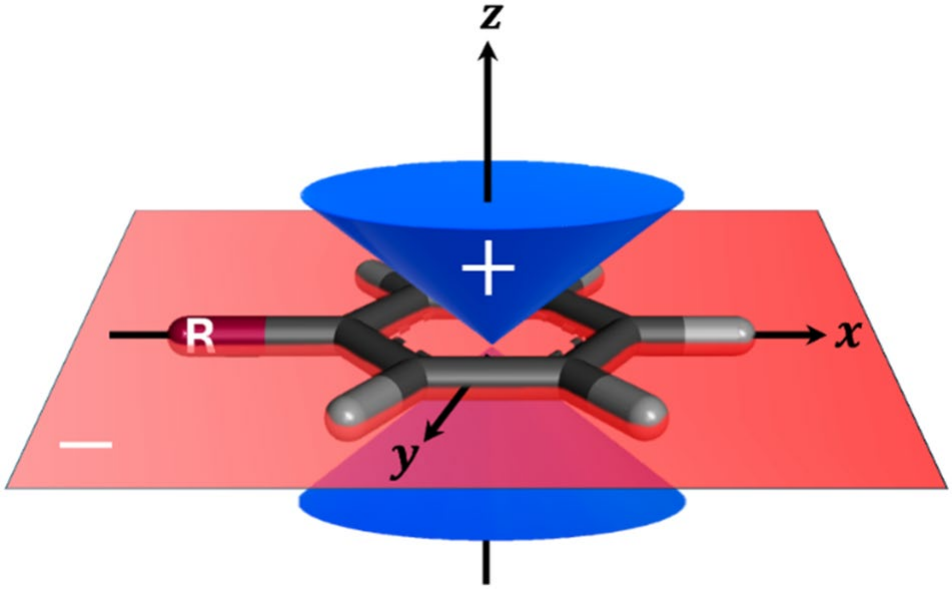
The angular factor $\left(\frac{3}{2}\cos^{2}\theta -\frac{1}{2}\right)$ of the $\Theta_{\mathrm{zz}}$ component of the $Q_{2}$ quadrupole tensor. The factor is positive for θ angles (with respect to the z‐axis) smaller than 54° and greater than 126° (blue cone), and negative near the *xy‐*plane (red region).

The $Q_{2}$‐based aromaticity descriptors involve out‐of‐plane components of the $Q_{2}$ tensor. For instance, the expectation value of the $\;\Theta_{\mathrm{zz}}$ component for a given states is calculated by Equation (1):

(1) $$\left\langle \left.\Theta_{\mathrm{zz}}\right\rangle \right.=\int \rho \left(r\right)r^{2}\left(\frac{3}{2}\cos^{2}\theta -\frac{1}{2}\right)dr$$

where $\rho \left(r\right)$ is the molecular charge density. The angular factor in parentheses of the quadrupole component $\Theta_{\mathrm{zz}}$ is positive when the polar angle $\theta <54.7^{{}^{\circ}}$ or $\theta >125.3^{{}^{\circ}}$, and is negative between these values in the region close to the xy plane. This angular factor as a function of θ (the angle with the *z* axis) is illustrated in Figure 2.

The Gaussian‐formatted checkpoint files (*.*fchk*) were then used to partition the electron density according to the distributed multipole analysis (DMA) electric multipole expansion using the GDMA2 software [42, 43, 58]. GDMA2 computes the required components of the DMA $Q_{2}$ tensor, which will be used to calculate the six different aromaticity descriptors [42]. The components of the quadrupole $Q_{2}$ tensors are in atomic units $ea_{0}^{2}$.

The complete computational protocol for obtaining the $Q_{2}$‐based aromaticity indices is described in detail in our previous publication [48]. The Python script used to compute the $Q_{2}$‐based descriptors is openly available in the GitHub and Zenodo (DOI: https://doi.org/10.5281/zenodo.15276211) repositories [59]. Table 1 summarizes the definitions of the six $Q_{2}$‐based aromaticity descriptors used in this work [48].

**TABLE 1:** Aromaticity descriptors based on the DMA $Q_{2}$ tensor components; the components of $Q_{2}$ tensor are given in atomic units ($ea_{0}^{2}$) [48].^a^

Aromaticity index	Definition
${\left|Q_{2}\right|}_{\text{ring atoms}}$	The total sum of the $\left|Q_{2}\right|$ values of each ring atom
${\left[Q\right]}_{2_{\mathrm{zz},\text{ring atoms}}}$	The total sum of the $Q_{20}=Q_{\mathrm{zz}}$ values of each ring atom
${\left|Q_{2}\right|}_{\text{origin}}$	The total sum of the $\left|Q_{2}\right|$values computed for all the atoms of a molecule referred to $r_{\text{origin}}=\left(0,0,0\right)$
${\left[Q\right]}_{2_{\mathrm{zz},\text{origin}}}$	The total sum of the $Q_{20}=Q_{\mathrm{zz}}$ values of all the atoms of a molecule referred to $r_{\text{origin}}=\left(0,0,0\right)$
$\left|Q_{2}\right|\left(1\right)$	The magnitude of $Q_{2}$ computed at $r=\left(0,0,1\;Å\right)$
$Q_{2}{\left(1\right)}_{\mathrm{zz}}$	The value of $Q_{20}=Q_{\mathrm{zz}}$ computed at $r=\left(0,0,1\;Å\right)$

*Note:* $Q_{\mathrm{zz}}$ is related to the out‐of‐plane component of the $Q_{2}$ tensor, being defined in the following way: $Q_{\mathrm{zz}}=Q_{20}=\int \rho \left(r\right)R_{20}\left(r\right)\mathrm{dr}=\mathbb{C}\int \rho \left(r\right)r^{2}\left(\frac{3}{2}\cos^{2}\theta -\frac{1}{2}\right)\mathrm{dr},$ where $\rho \left(r\right)$ is the molecular charge density, $R_{20}$ is the real spherical harmonic, θ is the angle with the z axis, and ℂ is a normalization constant.

^a^ Adapted from ACS Omega, 2025 (https://doi.org/10.1021/acsomega.4c11451) under the terms of the CC BY 4.0 license.

We employed the normalized (or relative) values of the $Q_{2}$‐based aromaticity descriptors. These normalized values were obtained by dividing the descriptor values of each compound by the corresponding values for benzene, which was used as a reference due to its well‐established aromatic character. In this work, only the normalized descriptor values will be discussed.

A clustering analysis was performed on the computed $Q_{2}$‐based aromaticity descriptors. First, all the $Q_{2}$‐based descriptor values were standardized via *z*‐score normalization. Principal‐Component Analysis (PCA) was then applied to the scaled data to reduce dimensionality to two orthogonal axes (PC1 and PC2) [60], which capture the majority of variance. PCA is a multivariate statistical technique that identifies a new set of orthogonal basis vectors, the principal components (PCs), which successively maximize the variance of the projected data. As each PC is a linear combination of the original variables weighted by the eigenvector coefficients, PCA both reduces dimensionality and decorrelates the dataset, facilitating the visualization. Subsequently, four‐cluster partitions were obtained using the machine learning agglomerative hierarchical clustering analysis (HCA) employing Ward's criterion [61, 62], which was applied to the complete, standardized dataset. HCA builds a hierarchy of nested clusters by iteratively merging the pair of clusters that minimizes the increase in within‐cluster variance. Cluster assignments were projected onto the PCA plane for visual inspection, and a corresponding dendrogram was generated to illustrate the hierarchical structure of the clusters.

We also determined the ionization potential (IP), electron affinity (EA), electronegativity (χ) [63, 64], chemical hardness (η) [63, 65], electrophilicity index (ω) [66], electron‐donating power ($\omega^{-}$) [67], and electron‐accepting power ($\omega^{+}$) [67] for each system. These quantities provide a comprehensive framework for assessing electronic stability, reactivity, and responses to charge perturbations. Formally, the IP is defined as the energy required to remove an electron from a neutral species in the gas phase, while the EA is the energy released when an additional electron is added to the neutral species. To determine the IP and EA energies, using the Equations (2) and (3) below, we adopted an adiabatic scheme in which the geometries of the corresponding cation and anion were separately optimized [68]. The molecular energies of the neutral and ionic species were calculated in the gas phase using the B3LYP/def2‐TZVP level of theory.

(2) $${\mathrm{IP}}_{\left(\text{adiabatic}\right)}=E_{\text{cation}}-E_{\text{neutral}}$$

(3) $${\mathrm{EA}}_{\left(\text{adiabatic}\right)}=E_{\text{neutral}}-E_{\text{anion}}$$

The electronegativity χ represents the tendency of a molecule to attract electron density [63, 64], whereas the hardness η measures the resistance of a chemical species to charge transfer [63, 65]. The electrophilicity index ω describes the energy stabilization of a system upon acquiring additional electronic charge from the environment [66]. The $\omega^{-}$ characterizes the ability of the molecule to donate electron density to its surroundings, and $\omega^{+}$ reflects the maximal capacity of the species to accept electron density [67]. Using IP and EA, these chemical reactivity parameters are derived using Equations ((4), (5), (6), (7), (8)) below.

(4) $$\chi =\frac{\mathrm{IP}+\mathrm{EA}}{2}$$

(5) $$\eta =\frac{1}{2}\left(\mathrm{IP}-\mathrm{EA}\right)$$

(6) $$\omega =\frac{{\left(\mathrm{IP}+\mathrm{EA}\right)}^{2}}{4\left(\mathrm{IP}-\mathrm{EA}\right)}$$

(7) $$\omega^{-}=\frac{{\left(3\mathrm{IP}+\mathrm{EA}\right)}^{2}}{16\left(\mathrm{IP}-\mathrm{EA}\right)}$$

(8) $$\omega^{+}=\frac{{\left(\mathrm{IP}+3\mathrm{EA}\right)}^{2}}{16\left(\mathrm{IP}-\mathrm{EA}\right)}$$

## 3 Results and Discussion

The interactions between the out‐of‐plane $p_{z}$ orbitals in benzene derivatives are directly related to the electron delocalization above and below the ring, significantly contributing to the stability and aromaticity of these molecules [69]. Thus, the $\left|Q_{2}\right|\left(z\right)$ and $Q_{2}{\left(z\right)}_{\mathrm{zz}}$ descriptors for benzene were evaluated at $r=\left(0,0,z\right)$ to find the distance that maximizes them. The $Q_{2}$ out‐of‐plane descriptors for benzene reach their maximum at z equal to 1 Å, as previously reported [48]. For this reason, the corresponding descriptors were defined at this distance.

We compared using scatter plots our $Q_{2}$‐based aromaticity descriptor values with the previous results on the same molecules that employed widely used descriptors [49]. The correlation plots of the HOMA (Figure S2), $\mathrm{AI}\left(\mathrm{vib}\right)$ (Figure S3), BVI (Figure S4), $I_{\text{ring}}$ (Figure S5), MCI (Figure S6) $\text{NICS}\left(0\right)$ (Figure S7), $\text{NICS}\left(1\right)$ (Figure S8), and $\text{NICS}{\left(1\right)}_{\mathrm{zz}}$ (Figure S9) descriptors are available in the Supporting Information. Figure S10 displays the positions where the $Q_{2}$‐based descriptors are evaluated relative to the molecular plane. The corresponding numerical results are shown in Table S1, while our $Q_{2}$‐based descriptor values are presented in Table S2.

The HOMA descriptor is a descriptor based on bond length alternation, while $\mathrm{AI}\left(\mathrm{vib}\right)$ is a vibrational descriptor derived from local vibrational force constants, thus providing insight into the relative bond strengths within the ring. Poor correlation was found between the HOMA and $\mathrm{AI}\left(\mathrm{vib}\right)$ descriptors with the $Q_{2}$‐based descriptors; the best results were for $Q_{2}{\left(1\right)}_{\mathrm{zz}}$, which reached a $R^{2}$ of 0.61 and 0.57, respectively. This poor correlation is not surprising given that both HOMA and $\mathrm{AI}\left(\mathrm{vib}\right)$ are structural descriptors while the $Q_{2}$ descriptors are electronic‐based. Furthermore, the HOMA index has low sensitivity to the electronic effects of substituents due to the high structural rigidity of the benzene ring [50, 51]. This minimal structural differentiation is consistent with the well‐known stability of the aromatic system in benzene from a reactivity perspective: the benzene ring tends to preserve its π‐electron structure in typical reactions, favoring substitution mechanisms over addition reactions [51, 70, 71].

The descriptors ${\left|Q_{2}\right|}_{\text{ring atoms}}$ and $Q_{2_{\mathrm{zz},\text{ring atoms}}}$ showed no correlation with HOMA and $\mathrm{AI}\left(\mathrm{vib}\right)$ ($R^{2}$ lower than 0.20). However, by summing up the quadrupolar contributions over the ring carbons for the $Q_{2}$ indices, they directly reflect the local π‐density anisotropy, mirroring, in electronic terms, the C—C bond alternation quantified by HOMA and the site‐specific vibrational force constants captured by $\mathrm{AI}\left(\mathrm{vib}\right)$. Therefore, although no correlation was obtained, the following trend was observed: the normalized structural descriptors HOMA and $\mathrm{AI}\left(\mathrm{vib}\right)$ (results between parentheses) are expected to approach 1 for substituted systems by: —CH_3_ (1.00 and 0.91), —NH_2_ (0.99 and 0.90), and —OH (1.00 and 0.92) [49]. These expectations are confirmed by the normalized $Q_{2}$‐based descriptors. For instance, the ${\left|Q_{2}\right|}_{\text{ring atoms}}$ and $Q_{2_{\mathrm{zz},\text{ring atoms}}}$ normalized values (relative to benzene) are equal to 1.00 and 0.95 for $C_{6}H_{5}CH_{3}$, 1.04 and 1.01 for $C_{6}H_{5}NH_{2}$, and 1.02 and 0.98 for $C_{6}H_{5}\mathrm{OH}$, respectively.

Furthermore, for the disubstituted systems, Chagas and coworkers observed that substituents with filled or unfilled *p* orbitals (—CH_2_, —NH, and —O) strongly interact with the π‐electron density and thus have a pronounced effect on the aromaticity indices [49]. They attributed this result to the increased quinoid character introduced by such substituents, which reduces the aromaticity. However, for the ${\left|Q_{2}\right|}_{\text{ring atoms}}$ and $Q_{2_{\mathrm{zz},\text{ring atoms}}}$ descriptors, a significant decrease was found only for the $C_{6}H_{4}{\left(O\right)}_{2}$ molecule, with values of 0.87 and 0.79, respectively. In contrast, the $Q_{2}{\left(1\right)}_{\mathrm{zz}}$ descriptor shows a marked reduction for the disubstituted species $C_{6}H_{4}{\left(CH_{2}\right)}_{2}$, $C_{6}H_{4}{\left(\mathrm{NH}\right)}_{2}$, and $C_{6}H_{4}{\left(O\right)}_{2}$, with values of 0.88, 0.84, and 0.80, respectively, which indicates their greater sensitivity to the electronic perturbations induced by these substituents.

For the charged monosubstituted benzene derivatives ($C_{6}H_{5}CH_{2}^{-}$, $C_{6}H_{5}NH^{-}$, $C_{6}H_{5}O^{-}$, $C_{6}H_{5}CH_{2}^{+}$, $C_{6}H_{5}NH^{+}$, and $C_{6}H_{5}O^{+}$), greater variation was found for the $Q_{2}$‐based descriptors. Chagas and collaborators reported a deviation exceeding 0.2 that of reference benzene (1.0), values consistent with our descriptors. This greater variation of the charged monosubstituted benzene derivatives relative to the benzene's values is due to the presence of filled $p_{z}$ orbitals in the electron‐donating substituents (—$CH_{2}^{-}$, —$NH^{-}$, and —$O^{-}$) and empty $p_{z}$ orbitals in the electron‐accepting substituents (—$CH_{2}^{+}$, —$NH^{+}$, and —$O^{+}$). Negatively charged substituents, which feature filled $p_{z}$ orbitals with appropriate symmetry perpendicular to the ring plane, can contribute to the electron delocalization in the ring's π‐system, thereby increasing the electron density. In contrast, positively charged substituents, with vacant $p_{z}$ orbitals, function as electron density acceptors, withdrawing charge from the ring along the C—X bond direction, which reduces π‐delocalization and weakens the aromatic character. However, while HOMA and $\mathrm{AI}\left(\mathrm{vib}\right)$ values remain below 1, our results indicate that positively charged molecules fall below 1, whereas negatively charged ones exceed this threshold. In this context, the descriptors employed in this work differentiated the positively and negatively charged substituents and their effects on the aromaticity.

This differentiation is not a real effect arising from aromaticity, but rather a mathematical one discussed above, which involves the angular factor in Equation (1). In Figure 2, it can be noted that when negatively charged substituents, such as —${\mathrm{CH}}_{2}^{-}$, are located in the plane of the ring (where the angular factor is negative), their contribution to $\Theta_{\mathrm{zz}}$ is positive (the product of two negative signs), thus increasing its value. In contrast, positively charged substituents, such as (—${\mathrm{CH}}_{2}^{+}$), located in the same region with a negative angular factor, contribute with negative values to $\Theta_{\mathrm{zz}}$. Consequently, the presence of charged groups in the *xy* plane can significantly increase $Q_{2}$‐based descriptors without reflecting a real increase in the aromatic character of the system.

For the topological descriptor $\mathrm{EL}F_{\pi }$, the literature employs the bifurcation values index (BVI), defined as $\mathrm{BVI}=1-∆\mathrm{BV}\left(\mathrm{EL}F_{\pi }\right)=1-\left(BV_{\max}-BV_{\min}\right)$ [49]. BVI quantifies the uniformity of the π‐electron delocalization. The maximum bifurcation value $BV_{\max}$ corresponds to the value at which the π density is already completely divided into six regions, one for each atom of the aromatic ring, whereas the minimum value $BV_{\min}$ represents the first division of the π‐density within the ring. Again, a weak correlation was observed between the $Q_{2}$‐based descriptors and BVI, with *R* ^*2*^ values of 0.63 for $Q_{2}{\left(1\right)}_{\mathrm{zz}}$ and $R^{2}$ lower than 0.50 for the other BV descriptors (see Figure S3). However, the same conclusions can be obtained for the BVI and the $Q_{2}$‐based descriptors. For instance, for the BVI descriptor, Chagas et al. found that the —$CH_{3}$, —$NH_{2}$, and —OH substituents have comparable indices to those of benzene due to the lack of available orbitals for mixing with the ring π orbitals. Similarly, the $Q_{2}{\left(1\right)}_{\mathrm{zz}}$ values for these molecules vary from 0.95 to 0.96.

Chagas et al. also employed the electronic descriptors $I_{\text{ring}}$ [72] and MCI [39, 73]. Given a monocyclic system composed of n atoms labeled $A_{1}$, $A_{2}$, …, $A_{n}$, and a specified cyclic ordering $A\;\left(=A_{1}\to A_{2}\to \ldots \to A_{n}\to A_{1}\right)$, the $I_{\text{ring}}$ descriptor treats the π‐electron cloud as if it were flowing once around the ring, stepping from one atom to its neighbor in the A ordering and back to the start [72]. Therefore, it measures how strongly electrons flow along that circuit. The MCI descriptor is a more complete version of $I_{\text{ring}}$, which considers every possible sequence of ordering of the $A_{n}$ atoms, thereby removing any bias from the choice of A ordering [39, 73]. The result is a single number that captures the ring's overall electron delocalization without depending on any particular path. A weak correlation was observed between the $Q_{2}{\left(1\right)}_{\mathrm{zz}}$ descriptors and both $I_{\text{ring}}$ and MCI, with *R* ^*2*^ values of 0.58 and 0.59, respectively, and a weaker correlation with the other $Q_{2}$‐based descriptors—see Figures S5a and S6a, respectively.

The NICS is a magnetic descriptor that evaluates the chemical shielding tensor at a specific point of the molecule and describes the relationship between the applied magnetic field and the induced magnetic field [30]. Given that it can be calculated at points outside the molecular plane, the $\text{NICS}{\left(1\right)}_{\mathrm{zz}}$ was proposed for the studied molecules [49], which similarly to $Q_{2}{\left(1\right)}_{\mathrm{zz}}$, is computed 1 Å above the plane. Both $\text{NICS}{\left(1\right)}_{\mathrm{zz}}$ and $Q_{2}{\left(1\right)}_{\mathrm{zz}}$ are a reliable criterion for assessing aromaticity because they reflect the behavior of π‐electrons, which are primarily responsible for this phenomenon. Although a direct correlation between $\text{NICS}{\left(1\right)}_{\mathrm{zz}}$ and $Q_{2}{\left(1\right)}_{\mathrm{zz}}$ is relatively weak (*R* ^2^ value of 0.50—Figure S9a), both descriptors lead to consistent conclusions regarding aromatic character.

Analysis of the $\text{NICS}{\left(1\right)}_{\mathrm{zz}}$ values reveals that benzene and electron‐donating substituents —$CH_{3}$, —$NH_{2}$, and —OH exhibit pronounced out‐of‐plane magnetic shielding, indicative of strong aromaticity [49]. Accordingly, these same substituents display the highest $Q_{2}{\left(1\right)}_{\mathrm{zz}}$ values among the neutral derivatives, further supporting their aromatic character. In contrast, systems with substituents that significantly perturb the π‐electron distribution, most notably the cation $C_{6}H_{5}O^{+}$, show deshielded out‐of‐plane components in the $\text{NICS}{\left(1\right)}_{\mathrm{zz}}$ descriptor, consistent with an antiaromatic behavior. Likewise, $C_{6}H_{5}O^{+}$ also exhibits the lowest $Q_{2}{\left(1\right)}_{\mathrm{zz}}$ value in the series.

In disubstituted derivatives, the $Q_{2}{\left(1\right)}_{\mathrm{zz}}$ values for $C_{6}H_{4}{\left(CH_{3}\right)}_{2}$, $C_{6}H_{4}{\left(NH_{2}\right)}_{2}$, and $C_{6}H_{4}{\left(\mathrm{OH}\right)}_{2}$ remain relatively high, though slightly lower than those of their monosubstituted counterparts. Conversely, the neutral disubstituted derivatives —${\left(CH_{2}\right)}_{2}$, —${\left(\mathrm{NH}\right)}_{2}$, and —${\left(O\right)}_{2}$ exhibit markedly reduced shielding in $\text{NICS}{\left(1\right)}_{\mathrm{zz}}$, indicating diminished aromaticity. Similarly, their $Q_{2}{\left(1\right)}_{\mathrm{zz}}$ values approach those observed for positively charged substituents. The main difference between these two descriptors is that, while $\text{NICS}{\left(1\right)}_{\mathrm{zz}}$ provides similar values for positively and negatively charged substituents, $Q_{2}{\left(1\right)}_{\mathrm{zz}}$ offers a discriminative power, more effectively distinguishing between these electronic effects. Again, this discrimination is the result of a mathematical effect, discussed earlier in this article.

**FIGURE 3:**
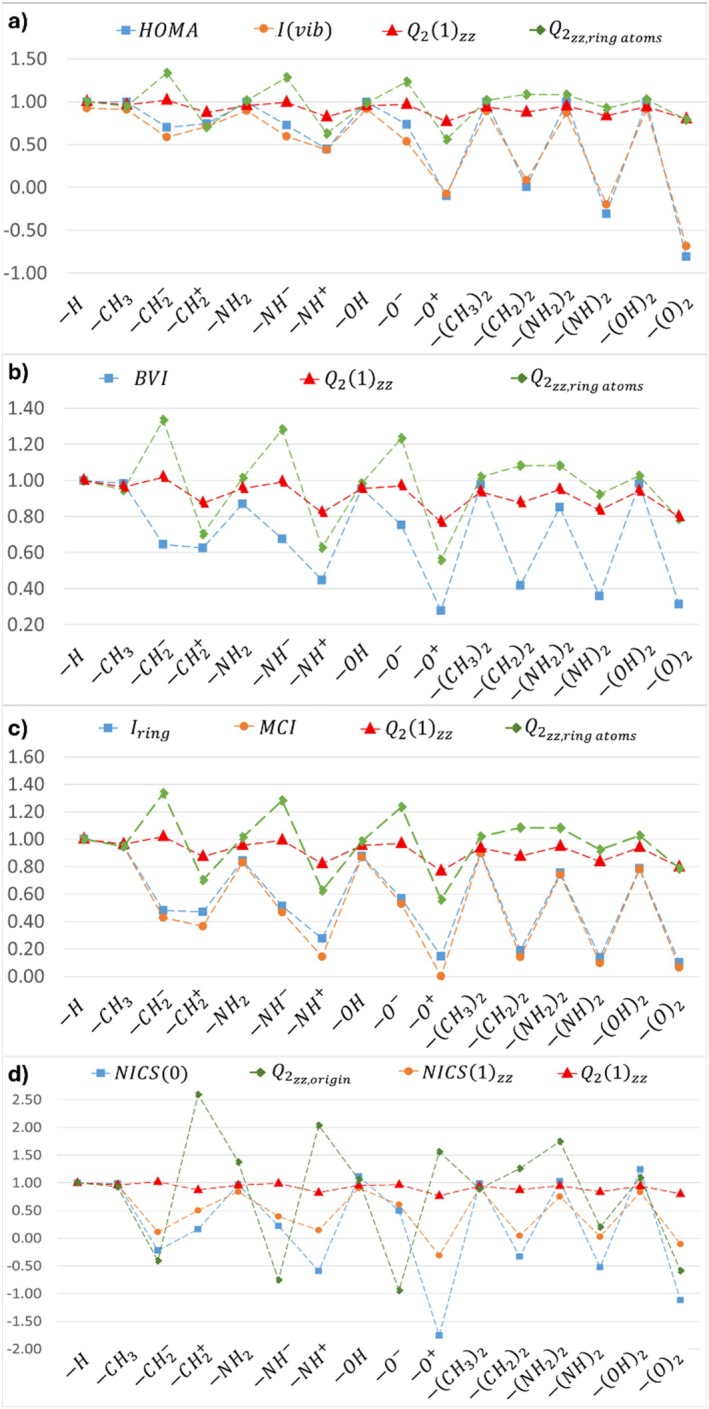
Comparison between the traditional aromaticity indices [49] and the $Q_{2}$‐based descriptors across the series of benzene derivatives. Traditional descriptors: (a) HOMA and $I\left(\mathrm{vib}\right)$; (b) BVI; (c) $I_{\text{ring}}$ and MCI; (d) $\text{NICS}\left(0\right)$, $\text{NICS}{\left(1\right)}_{\mathrm{zz}}$, all shown alongside the $Q_{2}{\left(1\right)}_{\mathrm{zz}}$ (red triangle) and $Q_{2_{\mathrm{zz},\text{origin}}}$ (green rhombus) values.

Overall, the results for the substituted benzene derivatives indicate that the correlations between the $Q_{2}$‐based descriptors and the others [49] are not significant. However, although no appreciable correlation was found, similar trends were found between those aromaticity descriptors (Figure 3a–d) and the $Q_{2}$‐based descriptors (Figure 3). For instance, it can be seen that although HOMA and $\mathrm{AI}\left(\mathrm{vib}\right)$ exhibit broader amplitude variations than the more narrowly constrained $Q_{2}$‐based descriptor (Figure 3a), all descriptors nonetheless follow the same relative patterns of increase and decrease across the substituent series. The exception to the trend arises for the $C_{6}H_{5}CH_{2}^{-}$, $C_{6}H_{5}NH^{-}$, and $C_{6}H_{5}O^{-}$ systems: while the values of the traditional descriptors [49] decrease along the series, the $Q_{2}{\left(1\right)}_{\mathrm{zz}}$ descriptor, for instance, increases. This discrepancy can be attributed to the negatively charged nature of the —$CH_{2}^{-}$, —$NH^{-}$, and —$O^{-}$ substituents. The same behavior (except for —$CH_{2}^{-}$, —$NH^{-}$, and —$O^{-}$) is observed for all the other aromaticity descriptors in this case (Figure 3). For these negatively charged substituents, it was found higher $Q_{2}$‐based descriptor values than for unsubstituted benzene, in contrast to the traditional indices, which tend to have lower values. This discrepancy is consistent with the mathematical definition of the out‐of‐plane quadrupole tensor components, which involves contributions from positive and negative charges in different regions of space, as discussed previously.

**FIGURE 4:**
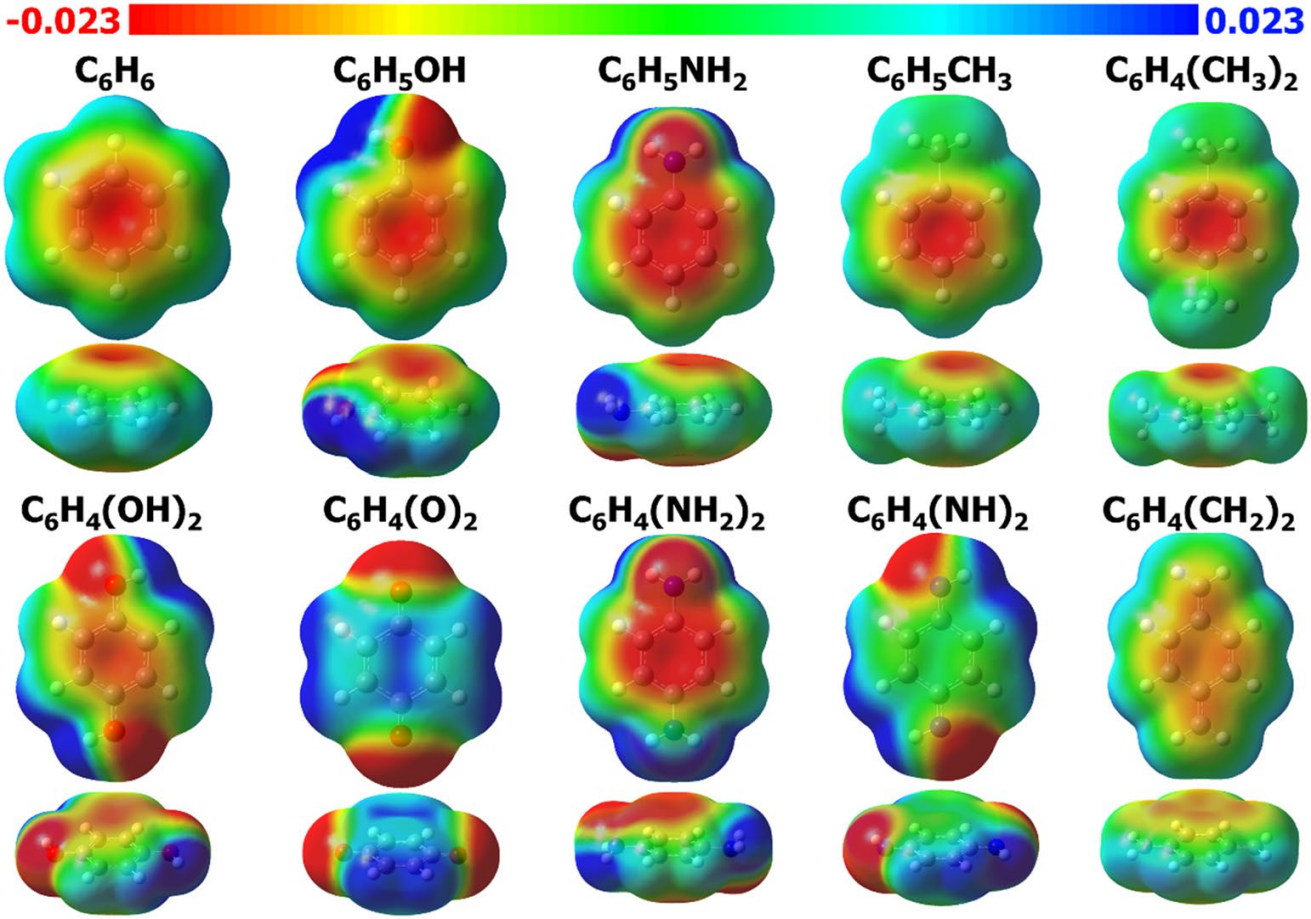
Molecular electrostatic potential maps for the neutral molecules (top and side views), shown for iso values of 0.001 a.u. Values given in $\text{kcal}\;\mathrm{mo}l^{-1}$.

**FIGURE 5:**
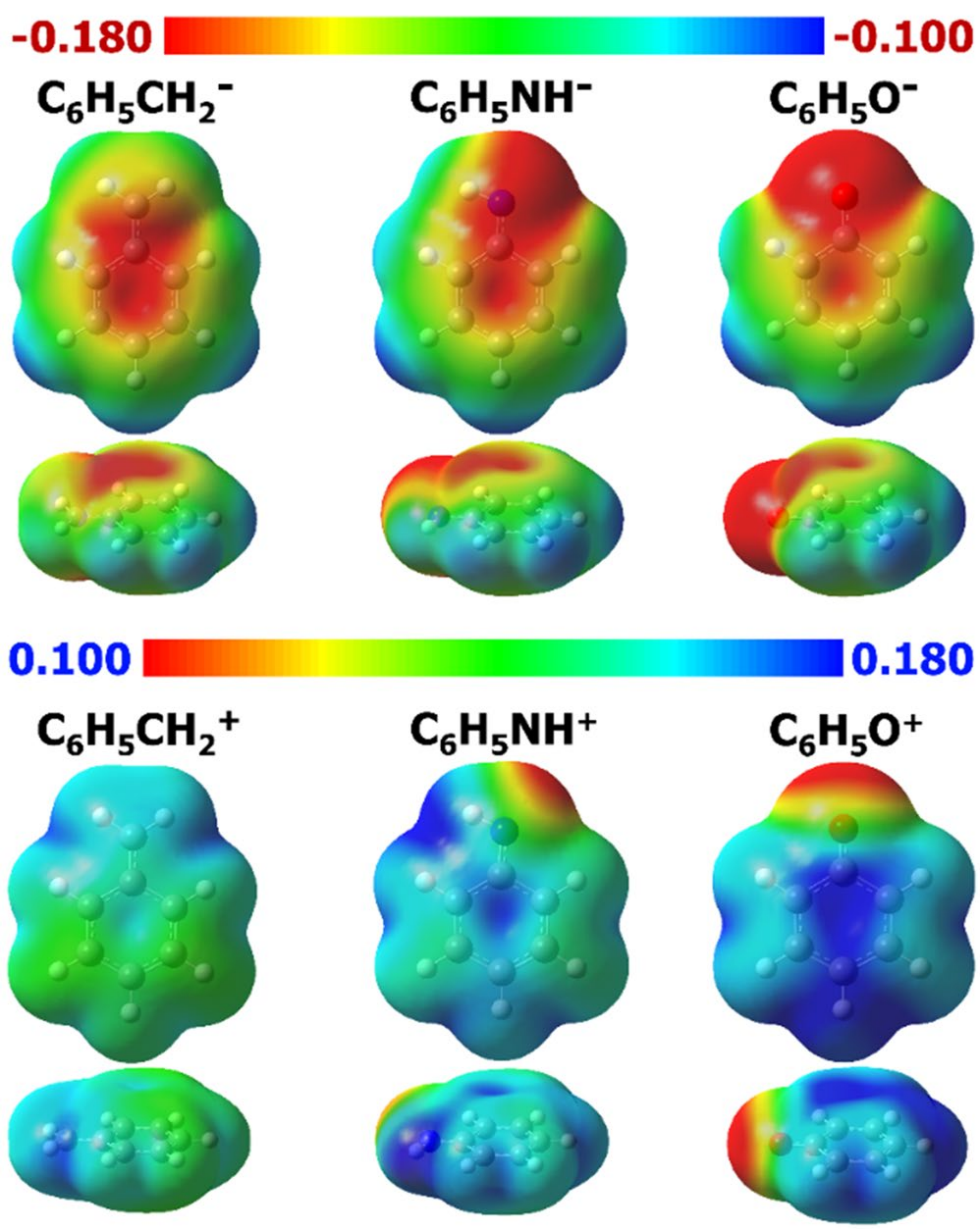
Molecular electrostatic potential maps for the charged molecules, shown for iso values of 0.001 a.u. Values given in $\text{kcal}\;\mathrm{mo}l^{-1}$.

The effects of the substituents can be visualized graphically through the molecular electrostatic potential (MEP) maps. Figures 4 and 5 display the MEP maps for the investigated neutral and charged species, respectively. MEP maps indicate regions of higher and lower electron density, thereby providing a complementary visual analysis for the quantitative discussion above. The red color indicates regions with higher electron density, while blue denotes regions with lower electron density.

When a substituent decreases the electron density at the ring, the aromaticity of the molecule decreases, and the corresponding descriptors are expected to have lower values. Analogously, an increased delocalization outwards the ring also contributes to reduced aromaticity. For example, for the $Q_{2}{\left(1\right)}_{\mathrm{zz}}$ descriptor, a qualitative analysis of MEPs suggests that, for the molecules depicted in Figure 4, benzene is expected to show the highest $Q_{2}{\left(1\right)}_{\mathrm{zz}}$ value due to its pronounced electron density concentration (red color) within the ring and its symmetric distribution in the other regions (yellow, green, and blue). Subsequently, $C_{6}H_{5}CH_{3}$, $C_{6}H_{5}NH_{2}$, $C_{6}H_{4}{\left(NH_{2}\right)}_{2}$, $C_{6}H_{5}\mathrm{OH}$, $C_{6}H_{4}{\left(CH_{3}\right)}_{2}$, and $C_{6}H_{4}{\left(\mathrm{OH}\right)}_{2}$ display similarly significant normalized $Q_{2}{\left(1\right)}_{\mathrm{zz}}$ values, ranging from 0.96 to 0.94, which is consistent with the large electron density concentrated at the ring center (red color). For the remaining neutral molecules, the differences become more patent as the color of the central region shifts from light red to yellow for $C_{6}H_{4}{\left(CH_{2}\right)}_{2}$, green for $C_{6}H_{4}{\left(\mathrm{NH}\right)}_{2}$, and finally blue for $C_{6}H_{4}O_{2}$. Upon examining the $Q_{2}{\left(1\right)}_{\mathrm{zz}}$values (see Table S2), the order follows the expected trend, namely: $C_{6}H_{6}$, $C_{6}H_{5}CH_{3}$, $C_{6}H_{5}NH_{2}$, $C_{6}H_{4}{\left(NH_{2}\right)}_{2}$, $C_{6}H_{5}\mathrm{OH}$, $C_{6}H_{4}{\left(CH_{3}\right)}_{2}$, $C_{6}H_{4}{\left(\mathrm{OH}\right)}_{2}$, $C_{6}H_{4}{\left(CH_{2}\right)}_{2}$, $C_{6}H_{4}{\left(\mathrm{NH}\right)}_{2}$, and $C_{6}H_{4}{\left(O\right)}_{2}$.

For the charged species (Figure 5), the interpretation is similar. The $Q_{2}{\left(1\right)}_{\mathrm{zz}}$ descriptor values follows the order: $C_{6}H_{5}CH_{2}^{-}>C_{6}H_{5}NH^{-}>C_{6}H_{5}O^{-}>C_{6}H_{5}CH_{2}^{+}>C_{6}H_{5}NH^{+}>C_{6}H_{5}O^{+}$. As the charge is more delocalized throughout the molecule, the variation in the electrostatic potential values is small. Considering an MEP ranging from −0.180 to $-0.100\;\text{kcal}\;\mathrm{mo}l^{-1}$, a larger electron density separation—that is, a greater difference between more negative regions (red in Figure 5) and less negative regions (blue in Figure 5)—is observed for $C_{6}H_{5}{\mathrm{CH}}_{2}^{-}$, $C_{6}H_{5}{\mathrm{NH}}^{-}$, and $C_{6}H_{5}O^{-}$, in ascending order, therefore these species display lower values of $Q_{2}{\left(1\right)}_{\mathrm{zz}}$ in the same order. Conversely, for the cationic species, considering a MEP ranging from +0.100 to $+0.180\;\text{kcal}\;\mathrm{mo}l^{-1}$, $C_{6}H_{5}CH_{2}^{+}$ displays a greenish region at the center, which changes to blue in $C_{6}H_{5}NH^{+}$ and becomes even bluer in $C_{6}H_{5}O^{+}$, with the corresponding decrease in $Q_{2}{\left(1\right)}_{\mathrm{zz}}$ following the same ordering. The $Q_{2}{\left(1\right)}_{\mathrm{zz}}$ values are collected on Table S2.

To complement the assessment provided by the MEP maps, a statistical method named Principal Component Analysis (PCA) [60] and the unsupervised machine learning technique Hierarchical Clustering Analysis (HCA) [61] were carried out on the complete set of $Q_{2}$‐based descriptors. The PCA was applied to project the high‐dimensional data onto two principal axes, which allowed the resulting HCA clusters to be rendered in a clear and interpretable format. The resulting graph is presented in Figure S1a. A dendrogram was constructed using HCA with Ward's linkage criterion, based on the pairwise Euclidean distances across all standardized descriptors. This dendrogram (Figure S1b) provides a hierarchical view of descriptor similarity, highlighting the nested relationships between species and enabling the identification of cluster boundaries.

The positively charged species (—$O^{+}$, —$NH^{+}$, and —$CH_{2}^{+}$ substituents) are grouped (Figure S1a), as are the negatively charged species (—$O^{-}$, —$NH^{-}$, and —$CH_{2}^{-}$ substituents). The disubstituted derivatives $C_{6}H_{4}{\left(\mathrm{NH}\right)}_{2}$ and $C_{6}H_{4}{\left(O\right)}_{2}$ are clustered as those exhibiting the lowest values of $Q_{2}{\left(1\right)}_{\mathrm{zz}}$ among the neutral derivatives, comparable to those of the positively charged species. Notably, as depicted in Figure 4, $C_{6}H_{4}{\left(\mathrm{NH}\right)}_{2}$ and $C_{6}H_{4}{\left(O\right)}_{2}$ are the only compounds that do not display an electron density (red region) concentrated at the center of the aromatic ring in the MEP maps. Instead, they show a predominantly green and blue distribution, respectively, indicating a reduced electron density in the ring. The neutral disubstituted $C_{6}H_{4}{\left(\mathrm{NH}\right)}_{2}$ and $C_{6}H_{4}{\left(O\right)}_{2}$ compounds feature a double bond between the aromatic ring and the substituents. The presence of these double bonds imposes localization of the π‐bond between the *ortho* and *meta* carbon atoms relative to the substituent, thereby inducing a quinoidal structure. Compared to single‐bonded substituents, this structural modification significantly perturbs the aromatic character of the central ring. Moreover, quinoidal distortion is associated with long‐range intramolecular charge transfer from the substituent group toward the quinoidal substructure's terminal position(s) [51, 74]. Consequently, substituents capable of promoting a quinoidal configuration act as dearomatizing agents [75]. As a result, such substituents lead to a marked decrease in the $Q_{2}{\left(1\right)}_{\mathrm{zz}}$ and $\left|Q_{2}\right|\left(1\right)$ values, approaching those observed for the positively charged species. This trend is confirmed by the dendrogram (Figure S1b), which indicates that these neutral compounds are clustered together but positioned near the branch containing the positively charged species, indicating partial similarity in their electronic behavior. The remaining substituents are grouped due to their smaller influence on the aromatic ring and, as a consequence, their similar $Q_{2}$‐descriptor value.

Finally, to assess and interpret the aromatic character of the investigated compounds, chemical reactivity parameters were examined, namely, ionization potential (IP), electron affinity (EA), electronegativity (χ), chemical hardness (η), electrophilicity index (ω), electron‐donating power ($\omega^{-}$), and electron‐accepting power ($\omega^{+}$). As aromaticity manifests itself as an electron delocalization, which profoundly affects a molecule's electronic stability, charge distribution, and response to electron gain or loss, the IP, EA, χ, η, ω, $\omega^{-}$, and $\omega^{+}$ parameters set naturally encode information about such delocalization.

Table S2 presents the coefficient of determination ($R^{2}$) obtained from the correlation between the IP, EA, χ, η, ω, $\omega^{-}$, and $\omega^{+}$ set with both $Q_{2}$‐based and the traditional aromaticity descriptors [49]. It can be seen that, except for ${\left|Q_{2}\right|}_{\text{origin}}$ and $Q_{2_{\mathrm{zz},\text{origin}}}$, the $Q_{2}$‐based descriptors correlate well with IP, EA, ω, $\omega^{-}$, and $\omega^{+}$. In contrast, BVI, $I_{\text{ring}}$, MCI, $\text{NICS}\left(0\right)$, $\text{NICS}\left(1\right)$, and $\text{NICS}{\left(1\right)}_{\mathrm{zz}}$ only correlates with η, while HOMA and $\mathrm{AI}\left(\mathrm{vib}\right)$ do not correlate with any of the reactivity descriptors.

**FIGURE 6:**
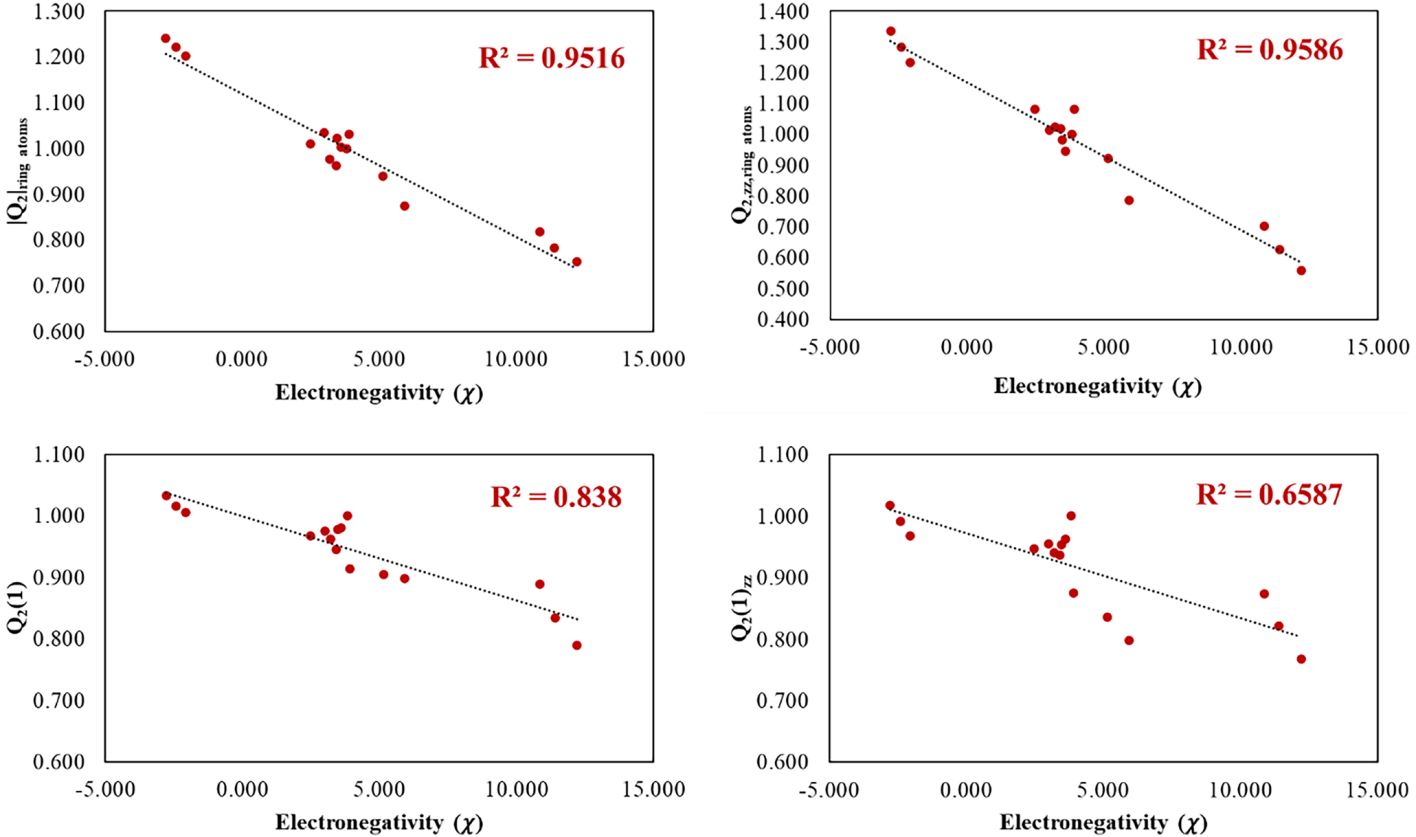
Correlation between electronegativity and the ${\left|Q_{2}\right|}_{\text{ring atoms}}$, $Q_{2_{\mathrm{zz},\text{ring atoms}}}$, $\left|Q_{2}\right|\left(1\right)$ and $Q_{2}{\left(1\right)}_{\mathrm{zz}}$ indices for the investigated molecules.

Given that reactivity is closely related to the stability of the aromatic ring, primarily due to the delocalization of π electrons, the presence of highly electronegative substituents decreases the electron distribution within the ring [22, 47]. The electron‐withdrawing effect of these substituents reduces the efficiency of π‐electron delocalization, thereby decreasing the aromatic character. The correlation observed between the descriptors ${\left|Q_{2}\right|}_{\text{ring atoms}}$, $Q_{2_{\mathrm{zz},\text{ring atoms}}}$, $\left|Q_{2}\right|\left(1\right)$ and $Q_{2}{\left(1\right)}_{\mathrm{zz}}$ and electronegativity (Figure 6) indicates that positively charged substituents, such as —$CH_{2}^{+}$, —$NH^{+}$, and —$O^{+}$, act as strong electron‐withdrawing groups: they exert a pronounced inductive effect, removing electron density from the ring through σ bonds, and additionally withdraw π‐electron density via resonance, with preferential delocalization from the ring toward the substituent. These combined effects reduce the electronic delocalization within the ring and, consequently, its aromaticity. In contrast, negatively charged substituents (—$CH_{2}^{-}$, —$NH^{-}$, and —$O^{-}$) exert an inductive electron‐donating effect through *σ* bonds, increasing the electron density in the ring and thus lowering its effective electronegativity, which favors electronic delocalization. The negative charge of these substituents enhances delocalization within the *π* system, therefore increasing electron density and accordingly modifying the aromaticity indices, as well as enhancing the ring's reactivity toward substitution reactions. Neutral substituents display an intermediate behavior between these two extremes.

Electrophilicity expresses the ability of a molecule to accept electrons during a chemical reaction. Similarly to the behavior for electronegativity, strongly electron‐withdrawing substituents—such as positively charged groups (—$CH_{2}^{+}$, —$NH^{+}$, and —$O^{+}$) or those with pronounced quinoid character (—${\left(CH_{2}\right)}_{2}$, —${\left(\mathrm{NH}\right)}_{2}$, and —${\left(O\right)}_{2}$)—exert an inductive effect that reduces the delocalization of π electrons in the aromatic ring. This decrease increases the electrophilicity of the system and, in turn, diminishes its aromatic character. Consistent with this reasoning, we observed that higher electrophilicity values correlate with lower values of the $Q_{2}$‐based descriptors (see Table S3). This behavior was also observed in our previous studies on systems substituted with fluorine atoms [47].

The correlations between IP, EA, χ, η, ω, $\omega^{-}$, and $\omega^{+}$ set and the $Q_{2}$‐based descriptors, in contrast to the poor or negligible correlations with the HOMA, $\mathrm{AI}\left(\mathrm{vib}\right)$, BVI, $I_{\text{ring}}$, MCI, $\text{NICS}\left(0\right)$, $\text{NICS}\left(1\right)$, and $\text{NICS}{\left(1\right)}_{\mathrm{zz}}$ [49] values, indicate that our $Q_{2}$‐based descriptors better capture π‐electron delocalization as reflected in the reactivity parameters. Therefore, the $Q_{2}$‐based descriptors emerge as a very effective aromaticity measure when aromatic character is probed via reactivity parameters.

Although the $Q_{2}$ descriptor values for systems containing charged substituents should be interpreted with caution due to the effect related to the angular factor in Equation (1), the observed correlation with electronegativity supports the interpretation that $Q_{2}$‐based descriptors provide a complementary tool for analyzing the aromatic character of substituted benzene systems. To achieve a broader and more robust assessment of our descriptors, additional studies are currently underway that involve other systems, including heteroatoms and antiaromatic frameworks.

## 4 Conclusions

In this work, we applied a set of aromaticity descriptors derived from the Distributed Multipole Analysis (DMA) developed by our group to a diverse set of neutral and charged mono‐ and para‐disubstituted benzene derivatives. The $Q_{2}$‐based descriptors are built from different components of the $Q_{2}$ DMA quadrupole electric moment tensor, the first term of the DMA multipole expansion having contributions from the out‐of‐plane electron density. We evaluated the sensitivity and robustness of these descriptors in capturing electronic modifications induced by electron‐donating and electron‐withdrawing groups. For this purpose, we investigated the correlation between the $Q_{2}$‐based descriptors and other aromaticity descriptors (HOMA, $\mathrm{AI}\left(\mathrm{vib}\right)$, NICS, ELF, and MCI) as well as with the nature of the substituents.

Among the $Q_{2}$‐based descriptors, the descriptor $Q_{2}{\left(1\right)}_{\mathrm{zz}}$, which quantifies the out‐of‐plane electron density at 1 Å above the center of the ring plane, where the π‐electron density is greatest, displayed the strongest correlation with the traditional aromaticity indices HOMA, $\mathrm{AI}\left(\mathrm{vib}\right)$, BVI, $I_{\text{ring}}$, MCI, $\text{NICS}\left(0\right)$, $\text{NICS}\left(1\right)$, and $\text{NICS}{\left(1\right)}_{\mathrm{zz}}$. However, this correlation, in the best cases, has an $R^{2}$ value close to 0.6. Overall, no appreciable correlation was found with the other $Q_{2}$‐based descriptors.

The lack of correlation between the $Q_{2}$‐based descriptors and the traditional aromaticity indices suggests that $Q_{2}$‐based descriptors capture features of aromaticity not accessible through the conventional methods, an expected outcome given the model‐dependent nature of the aromaticity concept. Nevertheless, $Q_{2}$‐based descriptors follow the same aromaticity trends observed in the traditional indices, presenting both increases and decreases in the descriptors' magnitudes. The exceptions to this trend are for the negatively charged substituents. This deviation highlights, in this case, the ability of $Q_{2}$‐based descriptors to distinguish between positively and negatively charged substituents. However, the apparent increase and decrease in aromaticity for the negatively and positively charged substituents, respectively, is a mathematical artifact. Because these substituents lie in a region where the angular factor tensor component is negative (see Figure 2), the negative charge contributes positively to the $\Theta_{\mathrm{zz}}$ quadrupole tensor component, and the positive charge contributes negatively, which is unrelated to an actual increase or decrease in aromaticity. Consequently, the presence of charged groups in the *xy* plane can significantly increase the $Q_{2}$‐based descriptor values without reflecting the real effect of the aromatic character of the system.

Furthermore, the $Q_{2}$‐based indices exhibit correlation with the global electronic reactivity parameters, such as IP, EA, χ, ω, $\omega^{-}$, and $\omega^{+}$, but not with η. In contrast, the traditional aromaticity descriptors BVI, $I_{\text{ring}}$, MCI, $\text{NICS}\left(0\right)$, $\text{NICS}\left(1\right)$, and $\text{NICS}{\left(1\right)}_{\mathrm{zz}}$ correlate solely with η, while HOMA and $\mathrm{AI}\left(\mathrm{vib}\right)$ show no significant correlation with any of the reactivity parameters. These findings indicate that the $Q_{2}$‐based approach to aromaticity not only reflects π‐electron delocalization but also encodes key aspects of global electron density distribution, thereby bridging aromaticity and frontier‐orbital‐based chemical reactivity.

Overall, our results highlight the enhanced sensitivity and effectiveness of $Q_{2}$‐based descriptors in capturing the electronic influence of substituents on aromatic systems. All six $Q_{2}$‐based descriptors reproduce similar aromaticity trends but with different sensitivities depending on the type of system. The $Q_{2}{\left(1\right)}_{\mathrm{zz}}$ descriptor shows the best agreement with the traditional indices, making it the most reliable for comparison with them (HOMA, NICS, BVI, MCI, $I_{\text{ring}}$, and $\mathrm{AI}\left(\mathrm{vib}\right)$) in studies of substituent effects, while $Q_{2_{\mathrm{zz},\text{ring atoms}}}$ better correlates with the chemical reactivity descriptors (IP, EA, χ, η, ω, $\omega^{-}$, and $\omega^{+}$), thus reflecting the local π‐density anisotropy at the ring carbons. Given that charged substituents can artificially affect the quadrupole tensor values, caution is recommended in these cases.

## Author Contributions

**Matheus Máximo‐Canadas:** investigation, data curation, formal analysis, visualization, first draft, writing – review and editing. **Nathália M. P. Rosa:** investigation, data curation, formal analysis, visualization, writing – review, and editing. **Itamar Borges Jr:** conceptualization, methodology, data curation, formal analysis, visualization, validation, funding acquisition, project administration, resources, supervision, writing – review, and editing.

## Conflicts of Interest

The authors declare no conflicts of interest.

## Supporting information


**Data S1:** Supporting Information.

## Data Availability

The data that support the findings of this study are openly available in Zenodo at https://doi.org/10.5281/zenodo.15882738. The Supporting Information provides a dataset of the $Q_{2}$‐based aromaticity descriptors derived from the (DMA) for all the substituted benzene derivatives. It is subdivided into four sections. Section 1 presents the results of the $Q_{2}$‐based descriptors and the other descriptor from the literature. Section 2 compares the $Q_{2}$‐based descriptor values with those available in the literature using scatter plots. Section 3 provides a theoretical background for the $Q_{2}$‐based aromaticity descriptors. Section 4 presents the *xyz* coordinates of the studied molecules.
