# Supplementary material for: Aromaticity of Substituted Benzene Derivatives Employing a New Set of Aromaticity Descriptors Based on the Partition of Electron Density
Source: J Comput Chem. 2025 Nov 2;46(29):e70257. doi: 10.1002/jcc.70257 (PMC12580489; doi:10.1002/jcc.70257)
Supplement: Supplementary file 1 — Data S1: Supporting Information. [file JCC-46-0-s001.docx]

**Supplementary Material**

**Aromaticity of substituted benzene derivatives employing a new set of aromaticity descriptors based on the partition of electron density**

Matheus Máximo-Canadas^a^, Nathália M. P. Rosa^a^, Itamar Borges Jr^a,*^

^a^*Departamento de Química, Instituto Militar de Engenharia (IME), Praça General Tibúrcio, 80, Rio de Janeiro, RJ 22290-270, Brasil*

^*^Email: [itamar@ime.eb.br](mailto:itamar@ime.eb.br)

Summary

[1. Aromaticity Descriptors Values 3](#_Toc209777010)

[TABLE S1: The aromaticity descriptors for mono- ($\boldsymbol{C}\mathbf{6}\boldsymbol{H}\mathbf{5}\boldsymbol{X}$) and disubstituted ($\boldsymbol{C}\mathbf{6}\boldsymbol{H}\mathbf{4}\boldsymbol{X}$) benzene. $-\boldsymbol{X}$ is the substituent. Results from Chagas and collaborators.^1^ All descriptors are unitless. 3](#_Toc209777011)

[TABLE S2: The $\boldsymbol{Q}\mathbf{2}$-based normalized aromaticity descriptors for mono- ($\boldsymbol{C}\mathbf{6}\boldsymbol{H}\mathbf{5}\boldsymbol{X}$) and disubstituted ($\boldsymbol{C}\mathbf{6}\boldsymbol{H}\mathbf{4}\boldsymbol{X}$) benzene. $-\boldsymbol{X}$ is the substituent. All descriptors are unitless. 4](#_Toc209777012)

[FIGURE S1: Hierarchical clustering analysis (HCA) of benzene derivatives based on their $\boldsymbol{Q}\mathbf{2}$-based aromaticity descriptors. (a) Principal Component Analysis (PCA) projection of the standardized descriptor space, color-coded by cluster assignments obtained via Ward’s method. (b) Corresponding dendrogram representing the hierarchical relationships among substituents, with relative distances reflecting Euclidean dissimilarities in the multivariate space. 5](#_Toc209777013)

[2. $\boldsymbol{DMA}$-based Aromaticity Descriptors $\boldsymbol{\times}$ Literature Descriptors Comparison 6](#_Toc209777014)

[FIGURE S2: Scatter plots comparing the $\boldsymbol{HOMA}$descriptor values^1^ and our $\boldsymbol{Q}\mathbf{2}$-based normalized aromaticity descriptors. 6](#_Toc209777015)

[FIGURE S3: Scatter plots comparison the $\boldsymbol{AI}(\boldsymbol{vib})$ descriptor values^1^ and our $\boldsymbol{Q}\mathbf{2}$-based normalized aromaticity descriptors. 7](#_Toc209777016)

[FIGURE S4: Scatter plots comparing the $\boldsymbol{BVI}$descriptor values^1^ and our $\boldsymbol{Q}\mathbf{2}$-based normalized aromaticity descriptors. 8](#_Toc209777017)

[FIGURE S5: Scatter plots comparing the $\boldsymbol{Iring}$descriptor values^1^ and our $\boldsymbol{Q}\mathbf{2}$-based normalized aromaticity descriptors. 9](#_Toc209777018)

[FIGURE S6: Scatter plots comparing the $\boldsymbol{MCI}$descriptor values^1^ and our $\boldsymbol{Q}\mathbf{2}$-based normalized aromaticity descriptors. 10](#_Toc209777019)

[FIGURE S7: Scatter plots comparing the $\boldsymbol{NICS}\mathbf{0}$ descriptor values^1^ and our $\boldsymbol{Q}\mathbf{2}$-based normalized aromaticity descriptors. 11](#_Toc209777020)

[FIGURE S8: Scatter plots comparing the $\boldsymbol{NICS}(\mathbf{1})$ descriptor values^1^ and our $\boldsymbol{Q}\mathbf{2}$-based normalized aromaticity descriptors. 12](#_Toc209777021)

[FIGURE S9: Scatter plots comparing the $\boldsymbol{NICS}\mathbf{1}\boldsymbol{ZZ}$ descriptor values^1^ and our $\boldsymbol{Q}\mathbf{2}$-based normalized aromaticity descriptors. 13](#_Toc209777022)

[TABLE S3: Coefficient of determination ($\boldsymbol{R}\mathbf{2}$) for the relationship between all the investigated aromaticity descriptors and the conceptual descriptors $\boldsymbol{IP}$, $\boldsymbol{EA}$, $\boldsymbol{\chi}$, $\boldsymbol{\eta}$, $\boldsymbol{\omega}$, $\boldsymbol{\omega}-$, and $\boldsymbol{\omega}+$. 14](#_Toc209777023)

[3. Theoretical Background of the $\boldsymbol{Q}\boldsymbol{2}$-Based Aromaticity Descriptors 15](#_Toc209777024)

[FIGURE S10: Schematic representation of the positions where the $\boldsymbol{Q}\mathbf{2}$-based descriptors are evaluated relative to the molecular plane. The blue point corresponds to $\boldsymbol{r} = (\mathbf{0}, \mathbf{0}, +\mathbf{1} Å)$, i.e., 1Å above the plane, related to $|\boldsymbol{Q}\mathbf{2}|(\mathbf{1})$and $\boldsymbol{Q}\mathbf{21}\boldsymbol{zz}$. The green point corresponds to the molecular origin, $\boldsymbol{rorigin}=(\mathbf{0}, \mathbf{0}, \mathbf{0})$, related to $\boldsymbol{Q}\mathbf{2}\boldsymbol{origin}$ and $\boldsymbol{Q}\mathbf{2}\boldsymbol{zz},\boldsymbol{origin}$. The red point corresponds to $\boldsymbol{r}=(\mathbf{0}, \mathbf{0}, -\mathbf{1} Å)$, i.e., 1Å below the plane, related to $|\boldsymbol{Q}\mathbf{2}|(-\mathbf{1})$and $\boldsymbol{Q}\mathbf{2}-\mathbf{1}\boldsymbol{zz}$. 16](#_Toc209777025)

[4. Coordinates 17](#_Toc209777026)

[TABLE S4: B3LYP/def2-TZVP Cartesian coordinates of the optimized molecules molecules from Chagas et al. ^1^. 17](#_Toc209777027)

[References 20](#_Toc209777028)

# 1. Aromaticity Descriptors Values

This section provides the numerical values of the aromaticity descriptors for the mono- ($C_{6}H_{5}X$) and disubstituted ($C_{6}H_{4}X$) benzene computed by Chagas *et al*. ^1^ It also presents the computed $\boldsymbol{Q}_{2}$-based descriptor values computed in this work.

## TABLE S1: The aromaticity descriptors for mono- ($\boldsymbol{C}_{\boldsymbol{6}}\boldsymbol{H}_{\boldsymbol{5}}\boldsymbol{X}$) and disubstituted ($\boldsymbol{C}_{\boldsymbol{6}}\boldsymbol{H}_{\boldsymbol{4}}\boldsymbol{X}$) benzene. $\boldsymbol{-X}$ is the substituent. Results from Chagas and collaborators.^1^ All descriptors are unitless.

| $-X$ | $HOMA$ | $AI(vib)$ | $BVI$ | $MCI$ | $NICS\left( 0 \right)$  $normalized$ | $NICS\left( 1 \right)$  $normalized$ | $NICS\left( 1 \right)_{zz}$  $normalized$ |
| --- | --- | --- | --- | --- | --- | --- | --- |
| $-H$ | 0.998 | 0.925 | 1.000 | 0.072 | 1.000 | 1.000 | 1.000 |
| $-CH_{3}$ | 0.997 | 0.907 | 0.982 | 0.068 | 0.992 | 0.985 | 0.962 |
| $-CH_{2}^{-}$ | 0.702 | 0.587 | 0.646 | 0.031 | -0.222 | 0.074 | 0.111 |
| $-CH_{2}^{+}$ | 0.743 | 0.709 | 0.625 | 0.026 | 0.162 | 0.581 | 0.502 |
| $-NH_{2}$ | 0.992 | 0.895 | 0.869 | 0.060 | 0.968 | 0.869 | 0.839 |
| $-NH^{-}$ | 0.724 | 0.593 | 0.675 | 0.034 | 0.219 | 0.359 | 0.390 |
| $-NH^{+}$ | 0.448 | 0.437 | 0.448 | 0.010 | -0.591 | 0.179 | 0.142 |
| $-OH$ | 0.997 | 0.917 | 0.955 | 0.062 | 1.115 | 0.945 | 0.907 |
| $-O^{-}$ | 0.736 | 0.537 | 0.753 | 0.038 | 0.497 | 0.566 | 0.606 |
| $-O^{+}$ | -0.100 | -0.076 | 0.278 | 0.000 | -1.754 | -0.391 | -0.312 |
| $-\left( CH_{3} \right)_{2}$ | 0.997 | 0.890 | 0.976 | 0.064 | 0.987 | 0.956 | 0.911 |
| $-\left( CH_{2} \right)_{2}$ | 0.006 | 0.085 | 0.418 | 0.010 | -0.332 | 0.137 | 0.045 |
| $-\left( NH_{2} \right)_{2}$ | 0.995 | 0.871 | 0.851 | 0.053 | 1.028 | 0.825 | 0.755 |
| $-\left( NH \right)_{2}$ | -0.309 | -0.200 | 0.359 | 0.007 | -0.528 | 0.089 | 0.024 |
| $-\left( OH \right)_{2}$ | 0.997 | 0.910 | 0.981 | 0.056 | 1.244 | 0.912 | 0.831 |
| $-{(O)}_{2}$ | -0.808 | -0.684 | 0.314 | 0.005 | -1.122 | -0.137 | -0.107 |

## TABLE S2: The $\boldsymbol{Q}_{\boldsymbol{2}}$-based normalized aromaticity descriptors for mono- ($\boldsymbol{C}_{\boldsymbol{6}}\boldsymbol{H}_{\boldsymbol{5}}\boldsymbol{X}$) and disubstituted ($\boldsymbol{C}_{\boldsymbol{6}}\boldsymbol{H}_{\boldsymbol{4}}\boldsymbol{X}$) benzene. $\boldsymbol{-X}$ is the substituent. All descriptors are unitless.

| $-\boldsymbol{X}$ | $\left\vert\boldsymbol{Q}_{\boldsymbol{2}} \right\vert_{\boldsymbol{ring atoms}}$ | $\boldsymbol{Q}_{\boldsymbol{2}_{\boldsymbol{zz,ring atoms}}}$ | $\left\vert\boldsymbol{Q}_{\boldsymbol{2}} \right\vert_{\boldsymbol{origin}}$ | $\boldsymbol{Q}_{\boldsymbol{2}_{\boldsymbol{zz,origin}}}$ | $\boldsymbol{\vert}\boldsymbol{Q}_{\boldsymbol{2}}\boldsymbol{\vert(1)}$ | $\boldsymbol{Q}_{\boldsymbol{2}}\left( \boldsymbol{1} \right)_{\boldsymbol{zz}}$ |
| --- | --- | --- | --- | --- | --- | --- |
| –𝐻 | 1.00 | 1.00 | 1.00 | 1.00 | 1.00 | 1.00 |
| –𝐶𝐻_3_ | 1.00 | 0.95 | 0.93 | 0.93 | 0.98 | 0.96 |
| $-CH_{2}^{-}$ | 1.24 | 1.33 | 1.74 | -0.41 | 1.03 | 1.02 |
| $-CH_{2}^{+}$ | 0.82 | 0.70 | 2.88 | 2.59 | 0.89 | 0.87 |
| $-NH_{2}$ | 1.04 | 1.01 | 1.55 | 1.37 | 0.98 | 0.96 |
| $-NH^{-}$ | 1.22 | 1.28 | 2.28 | -0.75 | 1.02 | 0.99 |
| $-NH^{+}$ | 0.78 | 0.63 | 2.28 | 2.04 | 0.83 | 0.82 |
| $-OH$ | 1.02 | 0.98 | 1.45 | 1.06 | 0.98 | 0.95 |
| $-O^{-}$ | 1.20 | 1.23 | 2.24 | -0.95 | 1.00 | 0.97 |
| $-O^{+}$ | 0.75 | 0.56 | 1.66 | 1.56 | 0.79 | 0.77 |
| $-\left( CH_{3} \right)_{2}$ | 0.96 | 1.02 | 0.90 | 0.89 | 0.94 | 0.94 |
| $-\left( CH_{2} \right)_{2}$ | 1.03 | 1.08 | 1.26 | 1.26 | 0.91 | 0.88 |
| $-\left( NH_{2} \right)_{2}$ | 1.01 | 1.08 | 2.52 | 1.75 | 0.97 | 0.95 |
| $-\left( NH \right)_{2}$ | 0.94 | 0.92 | 2.75 | 0.20 | 0.90 | 0.84 |
| $-\left( OH \right)_{2}$ | 0.98 | 1.03 | 2.53 | 1.09 | 0.96 | 0.94 |
| $-({O)}_{2}$ | 0.87 | 0.79 | 2.85 | -0.59 | 0.90 | 0.80 |


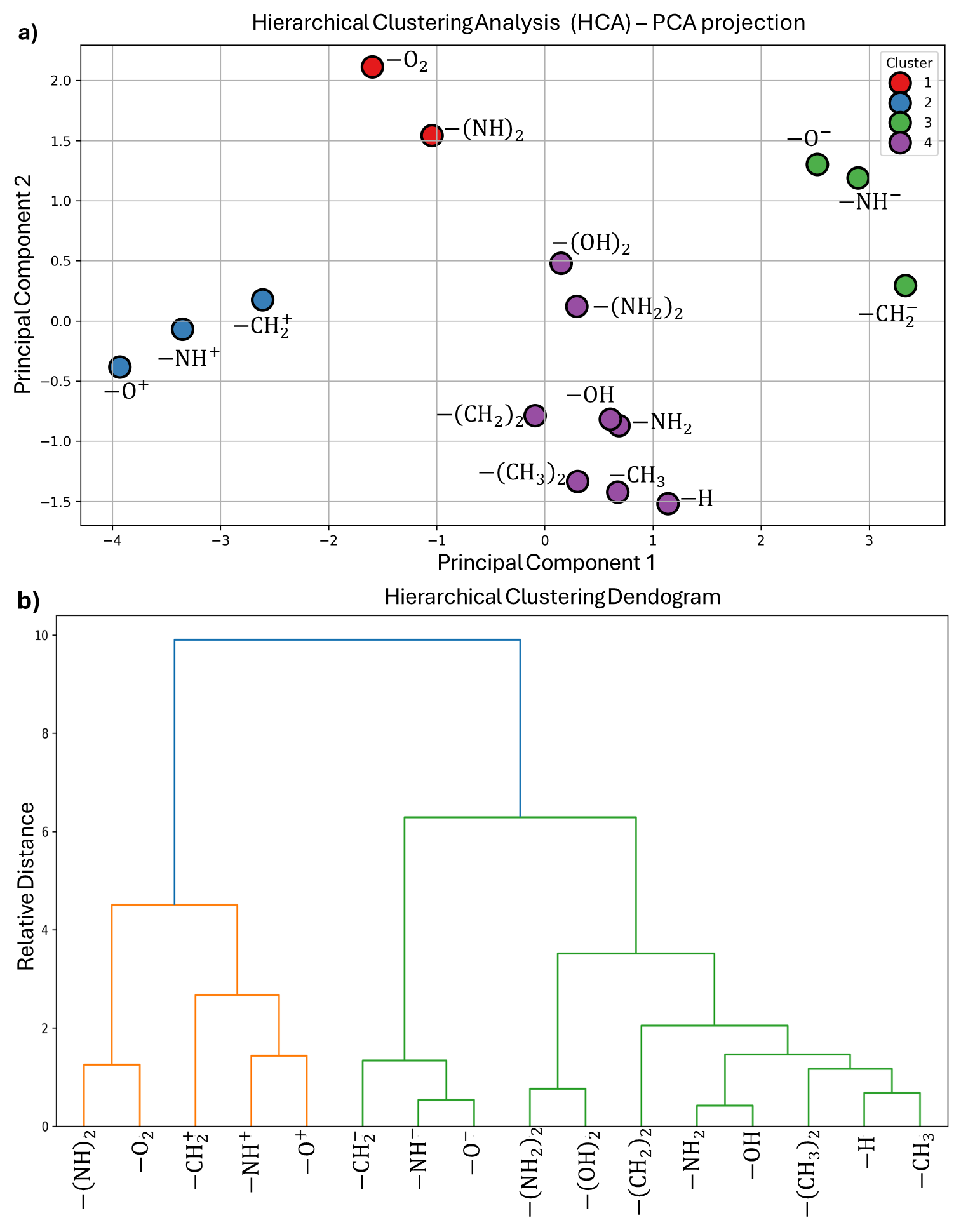


## FIGURE S1: Hierarchical clustering analysis (HCA) of benzene derivatives based on their $\boldsymbol{Q}_{\boldsymbol{2}}$-based aromaticity descriptors. (a) Principal Component Analysis (PCA) projection of the standardized descriptor space, color-coded by cluster assignments obtained via Ward’s method. (b) Corresponding dendrogram representing the hierarchical relationships among substituents, with relative distances reflecting Euclidean dissimilarities in the multivariate space.

# 2. $\boldsymbol{DMA}$-based Aromaticity Descriptors $\boldsymbol{\times}$ Literature Descriptors Comparison

This section provides the scatter plots to examine the correlation between the descriptor values from Chagas *et al.*^1^ and our $\boldsymbol{Q}_{2}$-based normalized aromaticity descriptors.


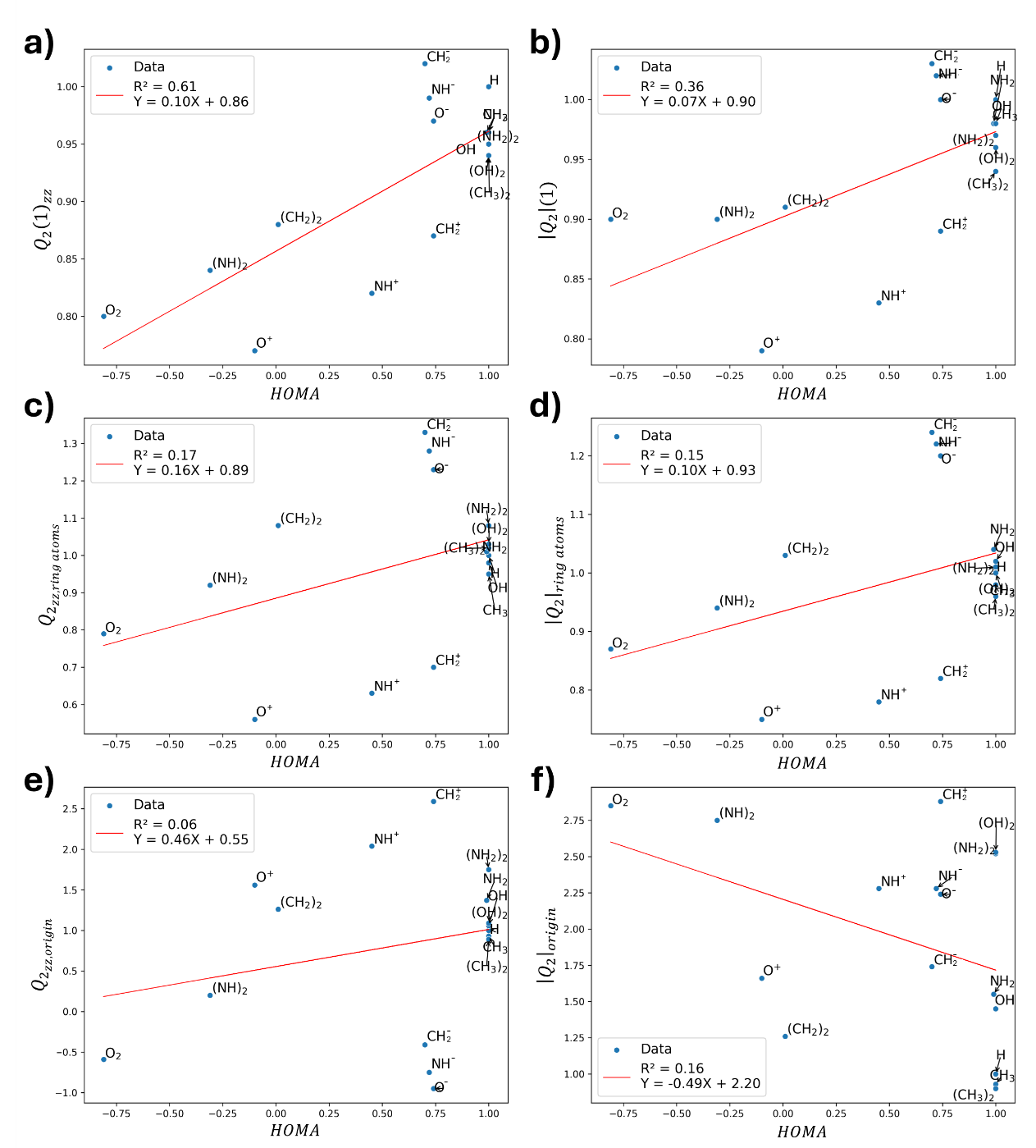


## FIGURE S2: Scatter plots comparing the $\boldsymbol{HOMA}$descriptor values^1^ and our $\boldsymbol{Q}_{\boldsymbol{2}}$-based normalized aromaticity descriptors.


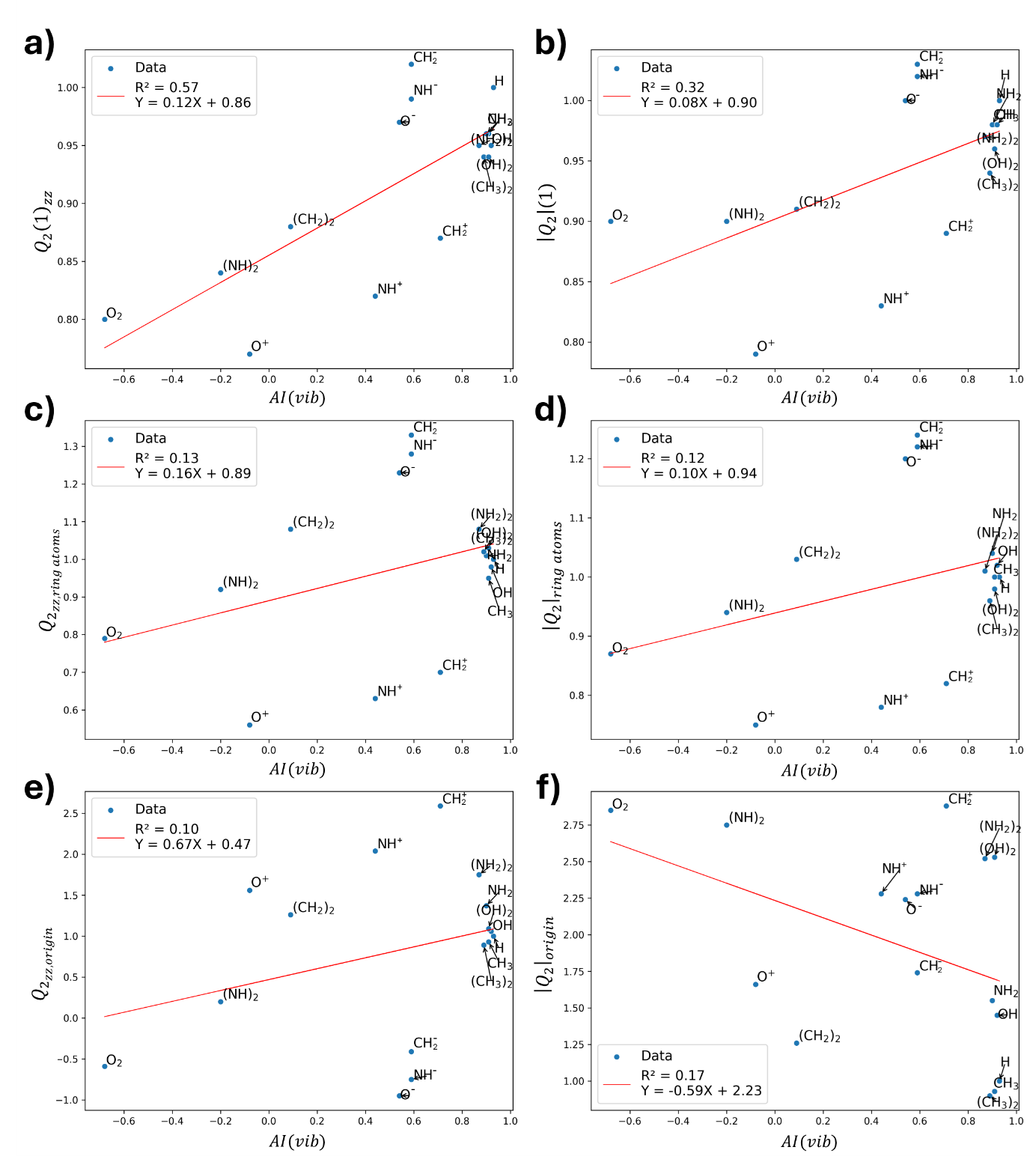


## FIGURE S3: Scatter plots comparison the $\boldsymbol{AI(vib)}$ descriptor values^1^ and our $\boldsymbol{Q}_{\boldsymbol{2}}$-based normalized aromaticity descriptors.


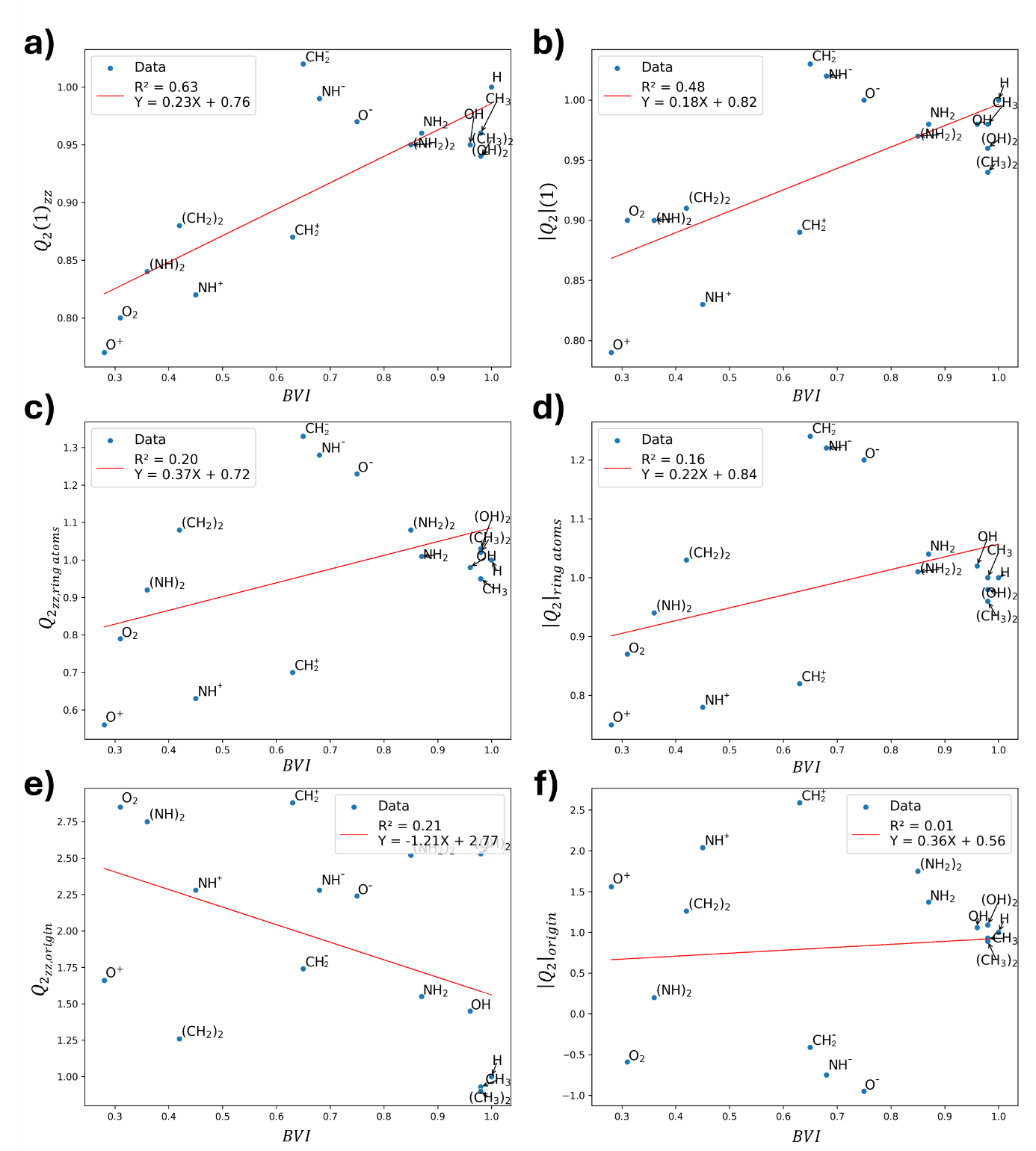


## FIGURE S4: Scatter plots comparing the $\boldsymbol{BVI}$descriptor values^1^ and our $\boldsymbol{Q}_{\boldsymbol{2}}$-based normalized aromaticity descriptors.


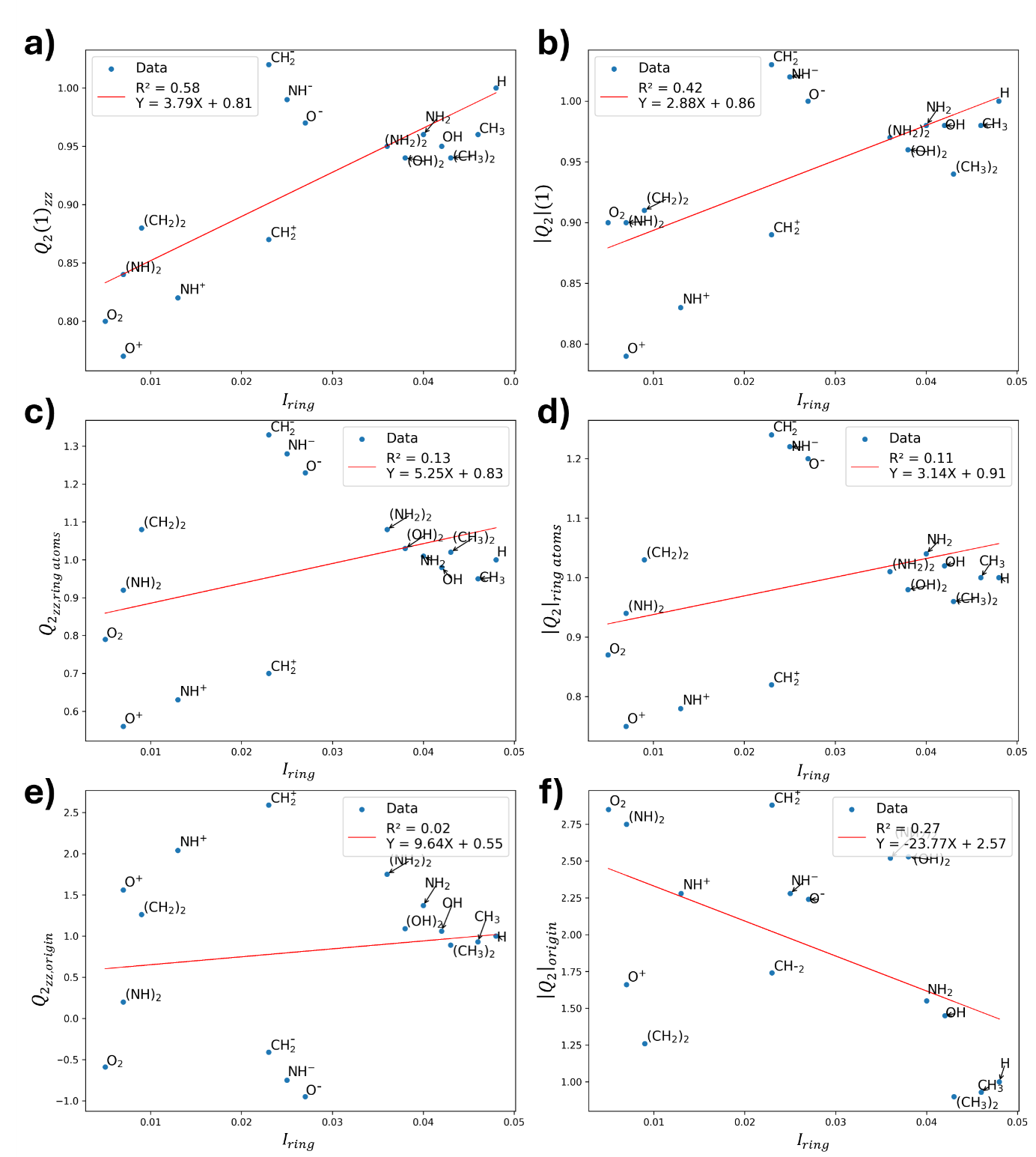


## FIGURE S5: Scatter plots comparing the $\boldsymbol{I}_{\boldsymbol{ring}}$descriptor values^1^ and our $\boldsymbol{Q}_{\boldsymbol{2}}$-based normalized aromaticity descriptors.


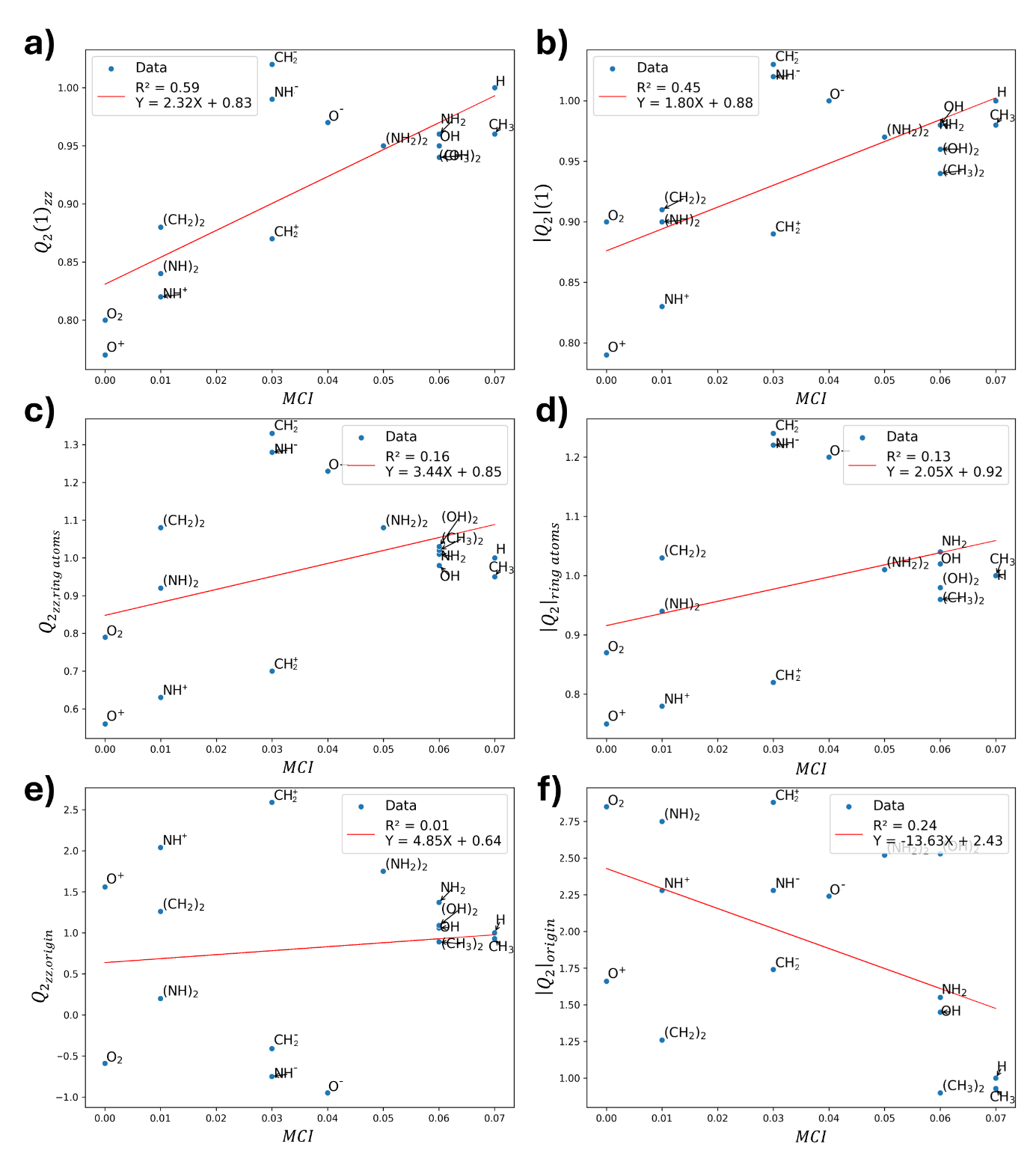


## FIGURE S6: Scatter plots comparing the $\boldsymbol{MCI}$descriptor values^1^ and our $\boldsymbol{Q}_{\boldsymbol{2}}$-based normalized aromaticity descriptors.


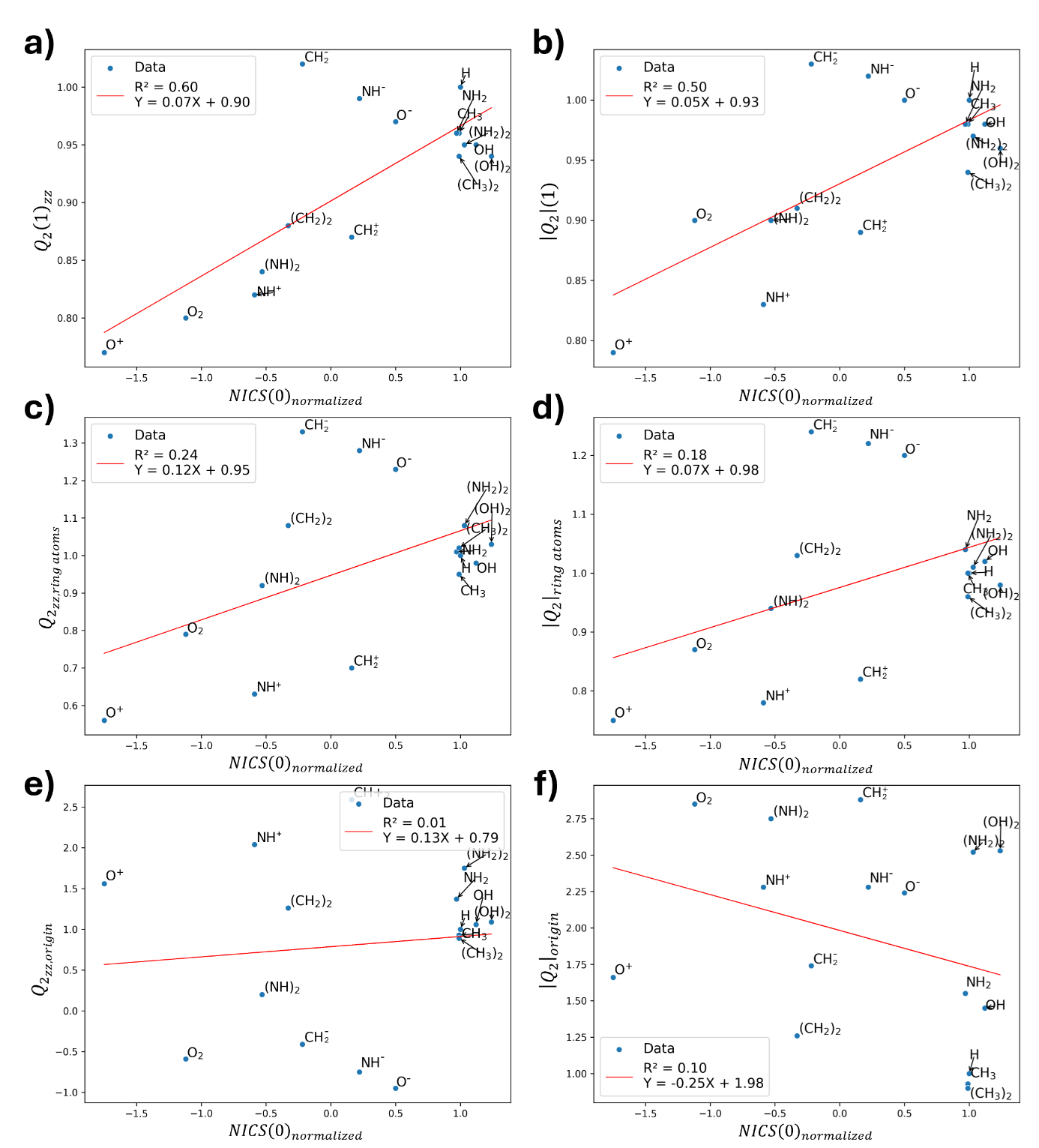


## FIGURE S7: Scatter plots comparing the $\boldsymbol{NICS}\left( \boldsymbol{0} \right)$ descriptor values^1^ and our $\boldsymbol{Q}_{\boldsymbol{2}}$-based normalized aromaticity descriptors.


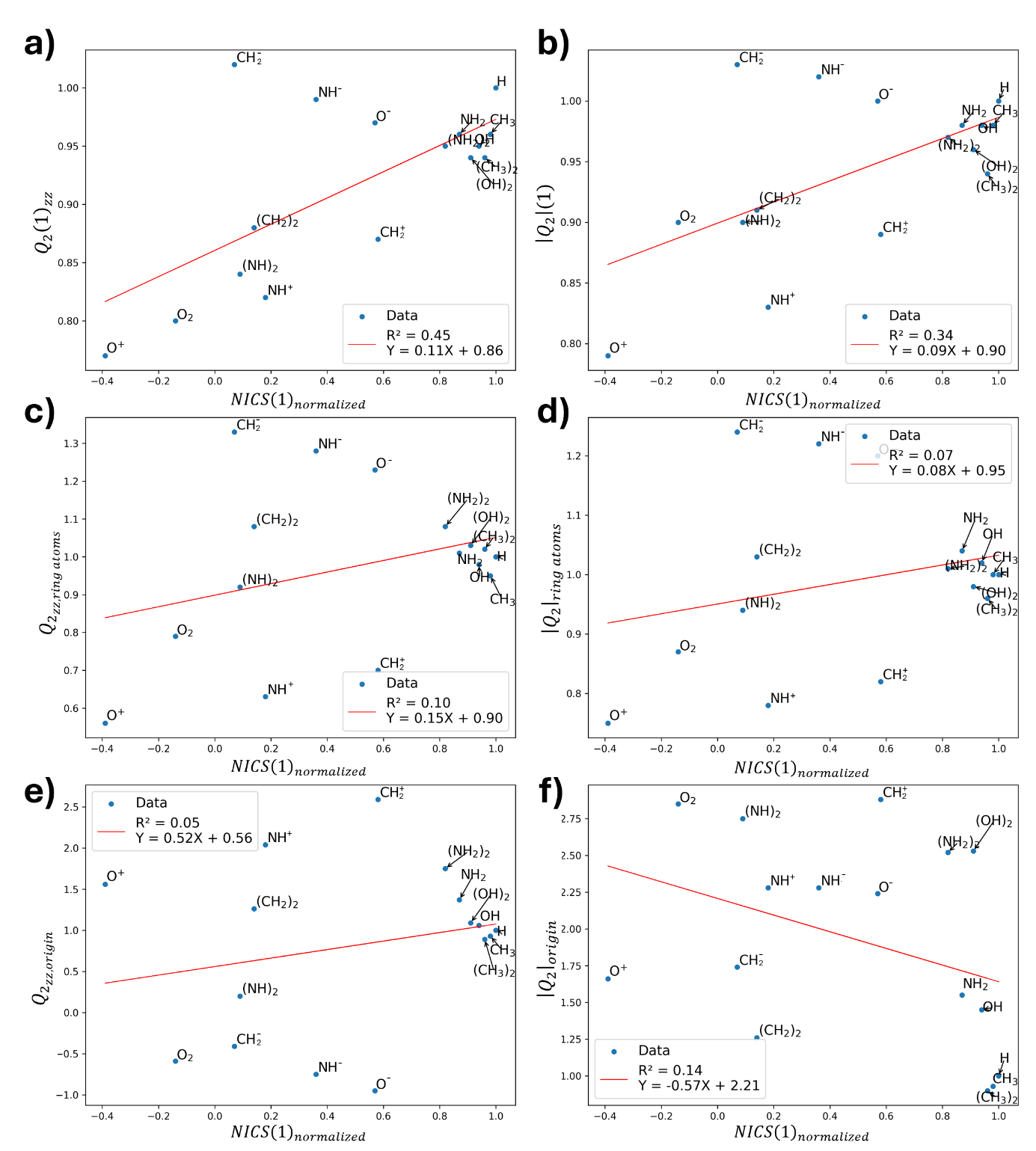


## FIGURE S8: Scatter plots comparing the $\boldsymbol{NICS(1)}$ descriptor values^1^ and our $\boldsymbol{Q}_{\boldsymbol{2}}$-based normalized aromaticity descriptors.


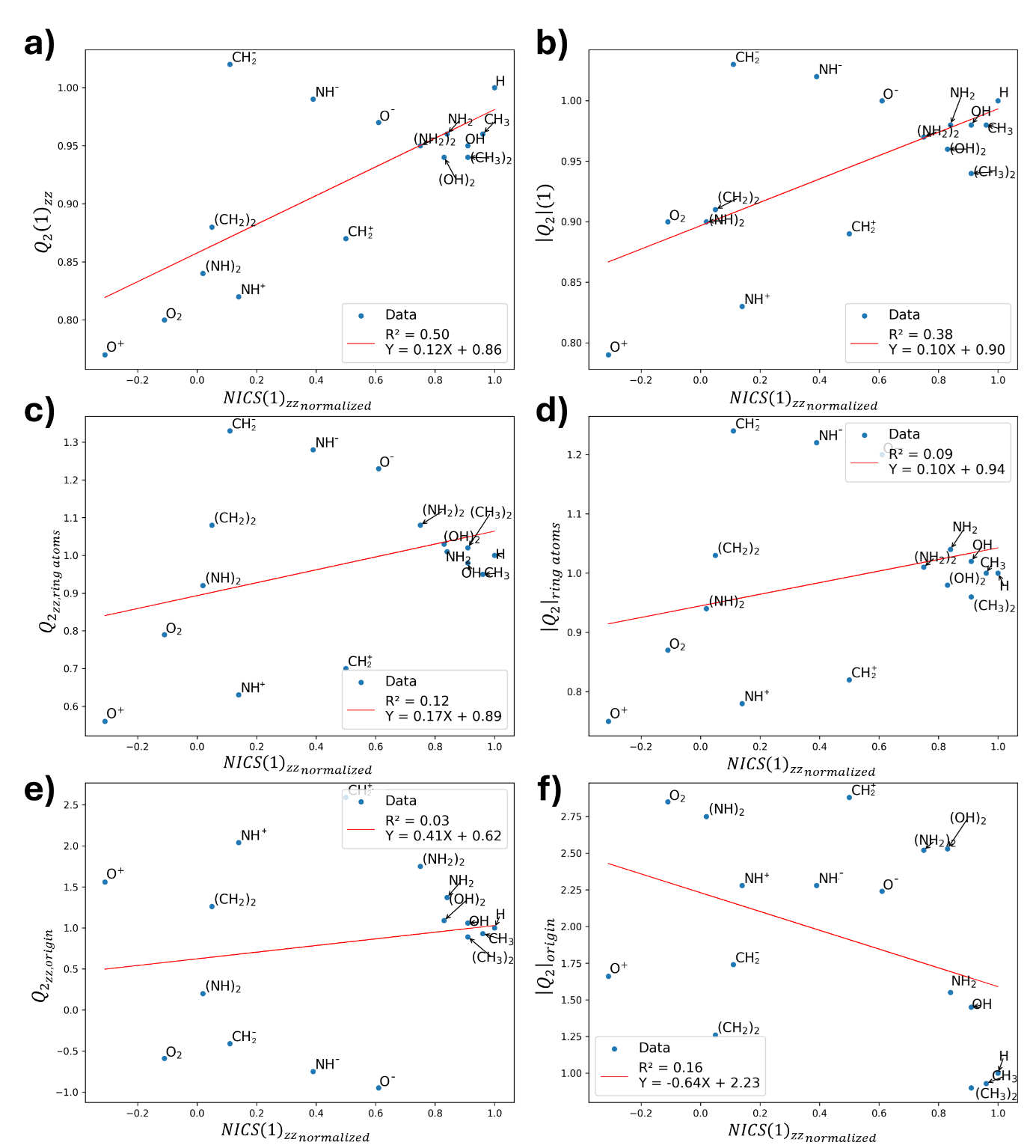


## FIGURE S9: Scatter plots comparing the $\boldsymbol{NICS}\left( \boldsymbol{1} \right)_{\boldsymbol{ZZ}}$ descriptor values^1^ and our $\boldsymbol{Q}_{\boldsymbol{2}}$-based normalized aromaticity descriptors.

## TABLE S3: Coefficient of determination ($\boldsymbol{R}^{\boldsymbol{2}}$) for the relationship between all the investigated aromaticity descriptors and the conceptual descriptors $\boldsymbol{IP}$, $\boldsymbol{EA}$, $\boldsymbol{\chi}$, $\boldsymbol{\eta}$, $\boldsymbol{\omega}$, $\boldsymbol{\omega}^{\boldsymbol{-}}$, and $\boldsymbol{\omega}^{\boldsymbol{+}}$.

|  | $\boldsymbol{IP}$ | $\boldsymbol{EA}$ | $\boldsymbol{\chi}$ | $\boldsymbol{\eta}$ | $\boldsymbol{\omega}$ | $\boldsymbol{\omega}^{\boldsymbol{-}}$ | $\boldsymbol{\omega}^{\boldsymbol{+}}$ |
| --- | --- | --- | --- | --- | --- | --- | --- |
| $\left\vert\boldsymbol{Q}_{\boldsymbol{2}} \right\vert_{\boldsymbol{ring atoms}}$ | 0.956 | 0.918 | 0.952 | 0.014 | 0.600 | 0.389 | 0.719 |
| $\boldsymbol{Q}_{\boldsymbol{2}_{\boldsymbol{zz,ring atoms}}}$ | 0.957 | 0.931 | 0.959 | 0.021 | 0.706 | 0.506 | 0.809 |
| $\left\vert\boldsymbol{Q}_{\boldsymbol{2}} \right\vert_{\boldsymbol{origin}}$ | 0.007 | 0.049 | 0.024 | 0.333 | 0.063 | 0.072 | 0.053 |
| $\boldsymbol{Q}_{\boldsymbol{2}_{\boldsymbol{zz,origin}}}$ | 0.543 | 0.484 | 0.521 | 0.000 | 0.289 | 0.171 | 0.360 |
| $\boldsymbol{\vert}\boldsymbol{Q}_{\boldsymbol{2}}\boldsymbol{\vert(1)}$ | 0.761 | 0.888 | 0.838 | 0.209 | 0.752 | 0.595 | 0.812 |
| $\boldsymbol{Q}_{\boldsymbol{2}}\left( \boldsymbol{1} \right)_{\boldsymbol{zz}}$ | 0.579 | 0.717 | 0.659 | 0.249 | 0.536 | 0.401 | 0.593 |
| $\boldsymbol{HOMA}$ | 0.088 | 0.175 | 0.131 | 0.299 | 0.115 | 0.087 | 0.123 |
| $\boldsymbol{AI(vib)}$ | 0.058 | 0.137 | 0.096 | 0.321 | 0.104 | 0.087 | 0.105 |
| $\boldsymbol{BVI}$ | 0.118 | 0.278 | 0.194 | 0.649 | 0.335 | 0.340 | 0.307 |
| $\boldsymbol{I}_{\boldsymbol{ring}}$ | 0.073 | 0.217 | 0.139 | 0.707 | 0.272 | 0.286 | 0.242 |
| $\boldsymbol{MCI}$ | 0.101 | 0.265 | 0.178 | 0.733 | 0.350 | 0.371 | 0.313 |
| $\boldsymbol{NICS(0)}$ | 0.148 | 0.299 | 0.222 | 0.528 | 0.420 | 0.443 | 0.380 |
| $\boldsymbol{NICS(1)}$ | 0.042 | 0.156 | 0.093 | 0.647 | 0.278 | 0.330 | 0.231 |
| $\boldsymbol{NICS}\left( \boldsymbol{1} \right)_{\boldsymbol{zz}}$ | 0.056 | 0.186 | 0.115 | 0.689 | 0.286 | 0.323 | 0.245 |

# 3. Theoretical Background of the $\boldsymbol{Q}_{\boldsymbol{2}}$-Based Aromaticity Descriptors

In this section, we briefly summarize the mathematical formulation of the $\boldsymbol{Q}_{2}$-based aromaticity descriptors. These descriptors were originally introduced in Ref. 2 by our group and are based on Stone’s distributed multipole analysis (DMA), which partition the molecular electron density into electric multipoles localized on different sites of a molecule. The $\boldsymbol{Q}_{2}$-based descriptors are derived from the different components of the DMA quadrupole electric moment tensor $\boldsymbol{Q}_{2}$, the first multipole term in the DMA expansion to the out-of-plane electron components.

The magnitude of the quadrupole moment tensor $\boldsymbol{Q}_{2}$ at a given point is given by:

| ${\vert\boldsymbol{Q}}_{2}\vert= \left( \sum_{k} \left\vert\boldsymbol{Q}_{2k} \right\vert^{2} \right)^{\frac{1}{2}}=\sqrt{{(Q_{20})}^{2}+{(Q_{21c})}^{2}+{(Q_{21s})}^{2}+{(Q_{22c})}^{2}+{(Q_{22s})}^{2}}$ | Eq. (1) |
| --- | --- |

where the tensor spherical components are expressed in terms of the Cartesian quadrupole tensor elements $Q_{20}$, $Q_{21c}$, $Q_{21s}$, $Q_{22c}$ and $Q_{22s}$. Their mathematical expressions are shown on Equation 2,

| $Q_{20}=Q_{2_{zz}}$, $Q_{21c}=\frac{2}{\sqrt{3}}\Theta_{xz}, Q_{21s}=\frac{2}{\sqrt{3}}\Theta_{yz}$,  $Q_{22s}=\frac{2}{\sqrt{3}}\Theta_{xy}$, $Q_{22c}=\frac{1}{\sqrt{3}}\left( \Theta_{xx}-\Theta_{yy} \right)$ | Eq. (2) |
| --- | --- |

Since π-electron delocalization in aromatic systems predominantly occurs above and below the molecular plane, it is particularly useful to consider descriptors that probe the electron density along the out-of-plane direction. In this context, a useful measure is obtained by summing the out-of-plane components of the quadrupole tensor ($Q_{2_{zz}}$).

From these definitions, a set of $\boldsymbol{Q}_{2}$-based aromaticity descriptors were constructed (defined in Table 1 of the manuscript). These descriptors includes values computed at the molecular origin ($\left| \boldsymbol{Q}_{2} \right|_{origin}$ and $Q_{2_{zz,origin}}$), the sum of the quadrupolar moment values of each ring atom ($\left| \boldsymbol{Q}_{2} \right|_{ring atoms}$ and $Q_{2_{zz,ring atoms}}$), or evaluated at positions 1Å above ($\left| \boldsymbol{Q}_{2} \right|(1)$ and $Q_{2}\left( 1 \right)_{zz}$) and below ($\left| \boldsymbol{Q}_{2} \right|(-1)$ and $Q_{2}\left( -1 \right)_{zz}$) the molecular plane. The latter are designed to minimize σ-density contributions and emphasize the π-electron delocalization that underlies aromaticity.


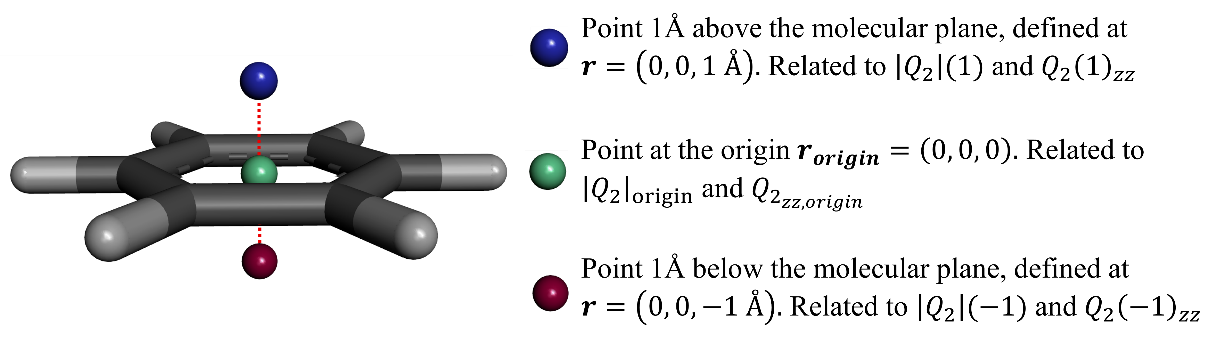


## FIGURE S10: Schematic representation of the positions where the $\boldsymbol{Q}_{\boldsymbol{2}}$-based descriptors are evaluated relative to the molecular plane. The blue point corresponds to $\boldsymbol{r = (0, 0, +1 Å)}$, i.e., 1Å above the plane, related to $\boldsymbol{|}\boldsymbol{Q}_{\boldsymbol{2}}\boldsymbol{|(1)}$and $\boldsymbol{Q}_{\boldsymbol{2}}\left( \boldsymbol{1} \right)_{\boldsymbol{zz}}$. The green point corresponds to the molecular origin, $\boldsymbol{r}_{\boldsymbol{origin}}\boldsymbol{=(0, 0, 0)}$, related to $\left| \boldsymbol{Q}_{\boldsymbol{2}} \right|_{\boldsymbol{origin}}$ and $\boldsymbol{Q}_{\boldsymbol{2}_{\boldsymbol{zz,origin}}}$. The red point corresponds to $\boldsymbol{r=(0, 0, -1 Å)}$, i.e., 1Å below the plane, related to $\boldsymbol{|}\boldsymbol{Q}_{\boldsymbol{2}}\boldsymbol{|(-1)}$and $\boldsymbol{Q}_{\boldsymbol{2}}\left( \boldsymbol{-1} \right)_{\boldsymbol{zz}}$.

All Q₂-based descriptors reported in this work are normalized with respect to the corresponding benzene value.

# 4. Coordinates

This section provides the xyz optimized coordinates for the studied molecules from Chagas *et al.* ^1^.

## TABLE S4: B3LYP/def2-TZVP Cartesian coordinates of the optimized molecules molecules from Chagas et al. ^1^.

| $\mathbf{C}_{\mathbf{6}}\mathbf{H}_{\mathbf{6}}$  C 0.00000000 1.20467177 0.69551377  C 0.00000000 0.00000000 1.39102632  C 0.00000000 -1.20467177 0.69551377  C 0.00000000 -1.20467177 -0.69551377  C 0.00000000 0.00000000 -1.39102632  C 0.00000000 1.20467177 -0.69551377  H 0.00000000 2.14253772 1.23699958  H 0.00000000 0.00000000 2.47398441  H 0.00000000 -2.14253772 1.23699958  H 0.00000000 -2.14253772 -1.23699958  H 0.00000000 0.00000000 -2.47398441  H 0.00000000 2.14253772 -1.23699958 | $\mathbf{C}_{\mathbf{6}}\mathbf{H}_{\mathbf{5}}\mathbf{C}\mathbf{H}_{\mathbf{3}}$  C 0.00427725 0.91099631 0.00000000  C 0.00734300 0.19349167 1.19730125  C 0.00734300 -1.19662177 1.20029486  C 0.00631594 -1.89799658 -0.00000000  C 0.00734300 -1.19662177 -1.20029486  C 0.00734300 0.19349167 -1.19730125  C -0.02810302 2.41726180 0.00000000  H 0.01224741 0.73041592 2.13919046  H 0.01200744 -1.73218916 2.14171530  H 0.00921256 -2.98067333 -0.00000000  H 0.01200744 -1.73218916 -2.14171530  H 0.01224741 0.73041592 -2.13919046  H -1.05761813 2.78755937 0.00000000  H 0.46436137 2.82632628 0.88334269  H 0.46436137 2.82632628 -0.88334269 |
| --- | --- |
| $\mathbf{C}_{\mathbf{6}}\mathbf{H}_{\mathbf{5}}\mathbf{C}\mathbf{H}_{\mathbf{2}}^{\mathbf{+}}$  C 0.00000000 0.00000000 -1.79437962  C 0.00000000 1.23044901 -1.11629352  C 0.00000000 1.24108004 0.25280749  C 0.00000000 0.00000000 0.97913438  C 0.00000000 -1.24108004 0.25280749  C 0.00000000 -1.23044901 -1.11629352  H 0.00000000 0.00000000 -2.87809572  H 0.00000000 2.15459644 -1.67761959  H 0.00000000 2.17268486 0.80452323  H 0.00000000 -2.17268486 0.80452323  H 0.00000000 -2.15459644 -1.67761959  C 0.00000000 0.00000000 2.34328792  H 0.00000000 0.92473686 2.90893239  H 0.00000000 -0.92473686 2.90893239 | $\mathbf{C}_{\mathbf{6}}\mathbf{H}_{\mathbf{5}}\mathbf{C}\mathbf{H}_{\mathbf{2}}^{\mathbf{-}}$  C 0.00000000 0.00000000 -1.87359840  C 0.00000000 1.19539407 -1.13609343  C 0.00000000 1.20828374 0.24234692  C 0.00000000 0.00000000 1.03742770  C 0.00000000 -1.20828374 0.24234692  C 0.00000000 -1.19539407 -1.13609343  H 0.00000000 0.00000000 -2.95686748  H 0.00000000 2.14678987 -1.66523341  H 0.00000000 2.16167914 0.76455731  H 0.00000000 -2.16167914 0.76455731  H 0.00000000 -2.14678987 -1.66523341  C 0.00000000 0.00000000 2.42187554  H 0.00000000 0.92653312 2.98447440  H 0.00000000 -0.92653312 2.98447440 |
| $\mathbf{C}_{\mathbf{6}}\mathbf{H}_{\mathbf{5}}\mathbf{N}\mathbf{H}_{\mathbf{2}}$  C 0.00238281 0.93537128 0.00000000  C 0.00308528 0.21991924 1.20312775  C 0.00308528 -1.16770899 1.19773958  C 0.00327083 -1.87500590 0.00000000  C 0.00308528 -1.16770899 -1.19773958  C 0.00308528 0.21991924 -1.20312775  N 0.06046324 2.32811227 0.00000000  H 0.00762686 0.75852110 2.14411494  H 0.00211452 -1.70017548 2.14104633  H 0.00254513 -2.95686736 0.00000000  H 0.00211452 -1.70017548 -2.14104633  H 0.00762686 0.75852110 -2.14411494  H -0.27661955 2.77733747 -0.83661613  H -0.27661955 2.77733747 0.83661613 | $\mathbf{C}_{\mathbf{6}}\mathbf{H}_{\mathbf{5}}\mathbf{N}\mathbf{H}^{\mathbf{+}}$  C 0.01278430 -1.76304419 0.00000000  C 1.24999109 -1.09093007 0.00000000  C 1.25984754 0.27355027 0.00000000  C 0.00000000 1.01238425 0.00000000  C -1.25711669 0.26870978 0.00000000  C -1.23371473 -1.09154121 0.00000000  H 0.01200744 -2.84767578 0.00000000  H 2.17036087 -1.65871990 0.00000000  H 2.17194807 0.85682686 0.00000000  N 0.08831621 2.29682198 0.00000000  H -2.18816867 0.82323111 0.00000000  H -2.14890880 -1.66796387 0.00000000  H -0.82620141 2.76177480 0.00000000 |
| $\mathbf{C}_{\mathbf{6}}\mathbf{H}_{\mathbf{5}}\mathbf{N}\mathbf{H}^{\mathbf{-}}$  C 0.02454445 -1.84506321 0.00000000  C 1.21425217 -1.09904300 0.00000000  C 1.21499143 0.27991286 0.00000000  C -0.00000000 1.06113414 0.00000000  C -1.20119584 0.25621411 0.00000000  C -1.17653382 -1.12629806 0.00000000  H 0.03583178 -2.92853243 0.00000000  H 2.16878090 -1.62203177 0.00000000  H 2.15106255 0.82941632 0.00000000  N 0.05304504 2.38655437 0.00000000  H -2.15798326 0.77452757 0.00000000  H -2.12152737 -1.66680947 0.00000000  H -0.90383029 2.74640814 0.00000000 | $\mathbf{C}_{\mathbf{6}}\mathbf{H}_{\mathbf{5}}\mathbf{OH}$  C 0.02168082 -1.85087446 0.00000000  C -1.18373444 -1.15997402 0.00000000  C -1.20047910 0.23039486 0.00000000  C 0.00000000 0.93680239 0.00000000  C 1.21293860 0.25180476 0.00000000  C 1.21625605 -1.13581879 0.00000000  H 0.03157977 -2.93274138 0.00000000  H -2.12123943 -1.70188591 0.00000000  H -2.14414699 0.76573750 0.00000000  O 0.04801839 2.30273909 0.00000000  H 2.13521714 0.81760803 0.00000000  H 2.16214984 -1.66306458 0.00000000  H -0.84767910 2.65842521 0.00000000 |
| $\mathbf{C}_{\mathbf{6}}\mathbf{H}_{\mathbf{5}}\mathbf{O}^{\mathbf{+}}$  C 0.00000000 0.00000000 -1.73768605  C 0.00000000 1.25414149 -1.07344801  C 0.00000000 1.28098457 0.28272873  C 0.00000000 0.00000000 1.04611110  C 0.00000000 -1.28098457 0.28272873  C 0.00000000 -1.25414149 -1.07344801  H 0.00000000 0.00000000 -2.82329964  H 0.00000000 2.16384939 -1.65924881  H 0.00000000 2.19545929 0.86376005  H 0.00000000 -2.19545929 0.86376005  H 0.00000000 -2.16384939 -1.65924881  O 0.00000000 0.00000000 2.25654478 | $\mathbf{C}_{\mathbf{6}}\mathbf{H}_{\mathbf{5}}\mathbf{O}^{\mathbf{-}}$  C 0.00000000 0.00000000 -1.82338182  C 0.00000000 1.19678129 -1.09762593  C 0.00000000 1.20911977 0.28612463  C 0.00000000 0.00000000 1.07729702  C 0.00000000 -1.20911977 0.28612463  C 0.00000000 -1.19678129 -1.09762593  H 0.00000000 0.00000000 -2.90742241  H 0.00000000 2.14385394 -1.63452482  H 0.00000000 2.14975268 0.82930188  H 0.00000000 -2.14975268 0.82930188  H 0.00000000 -2.14385394 -1.63452482  O 0.00000000 0.00000000 2.34154909 |
| $\mathbf{C}_{\mathbf{6}}\mathbf{H}_{\mathbf{4}}\left( \mathbf{C}\mathbf{H}_{\mathbf{3}} \right)_{\mathbf{2}}$  C -0.01091327 1.41734015 0.00248065  C -0.00620903 0.69389778 -1.19184232  C 0.00620903 -0.69389778 -1.19184232  C 0.01091327 -1.41734015 0.00248065  C 0.00616109 -0.69547983 1.19410760  C -0.00616109 0.69547983 1.19410760  H -0.01108426 1.22545047 -2.13709593  H 0.01108426 -1.22545047 -2.13709593  H 0.01086030 -1.22661867 2.13932447  H -0.01086030 1.22661867 2.13932447  C 0.00616109 2.92373582 -0.00234330  C -0.00616109 -2.92373582 -0.00234330  H -0.64189072 3.32726420 -0.78269411  H -0.32586034 3.32784847 0.95469869  H 1.01377060 3.30699773 -0.18864887  H 0.64189072 -3.32726420 -0.78269411  H 0.32586034 -3.32784847 0.95469869  H -1.01377060 -3.30699773 -0.18864887 | $\mathbf{C}_{\mathbf{6}}\mathbf{H}_{\mathbf{4}}\left( \mathbf{C}\mathbf{H}_{\mathbf{2}} \right)_{\mathbf{2}}$  C 0.00000000 0.00000000 1.44255674  C 0.00000000 1.23576101 0.67162412  C 0.00000000 1.23576101 -0.67162412  C 0.00000000 0.00000000 -1.44255674  C 0.00000000 -1.23576101 -0.67162412  C 0.00000000 -1.23576101 0.67162412  C 0.00000000 0.00000000 2.79118410  H 0.00000000 2.17131457 1.21884313  H 0.00000000 2.17131457 -1.21884313  C 0.00000000 0.00000000 -2.79118410  H 0.00000000 -2.17131457 -1.21884313  H 0.00000000 -2.17131457 1.21884313  H 0.00000000 -0.92363598 -3.35516023  H 0.00000000 0.92363598 -3.35516023  H 0.00000000 0.92363598 3.35516023  H 0.00000000 -0.92363598 3.35516023 |
| $\mathbf{C}_{\mathbf{6}}\mathbf{H}_{\mathbf{4}}\left( \mathbf{N}\mathbf{H}_{\mathbf{2}} \right)_{\mathbf{2}}$  C 0.00068964 1.41618356 0.00000000  C -0.00068964 0.69391873 1.19497899  C 0.00068964 -0.69391873 1.19497899  C -0.00068964 -1.41618356 0.00000000  C 0.00068964 -0.69391873 -1.19497899  C -0.00068964 0.69391873 -1.19497899  H 0.31054592 3.25185986 0.82986429  H 0.31054592 3.25185986 -0.82986429  H -0.00752106 1.22419096 2.14078682  H 0.00752106 -1.22419096 2.14078682  N 0.06772003 -2.82022536 0.00000000  H 0.00752106 -1.22419096 -2.14078682  H -0.00752106 1.22419096 -2.14078682  N -0.06772003 2.82022536 -0.00000000  H -0.31054592 -3.25185986 0.82986429  H -0.31054592 -3.25185986 -0.82986429 | $\mathbf{C}_{\mathbf{6}}\mathbf{H}_{\mathbf{4}}\left( \mathbf{NH} \right)_{\mathbf{2}}$  C 1.22550535 -0.75565244 0.00000000  C 1.22550535 0.71083449 0.00000000  C 0.08607210 1.41388950 0.00000000  C -1.22550535 0.75565244 0.00000000  C -1.22550535 -0.71083449 0.00000000  C -0.08607210 -1.41388950 0.00000000  N 2.35555472 -1.36523049 0.00000000  H 2.19682918 1.18866400 0.00000000  H 0.09918706 2.49836280 0.00000000  N -2.35555472 1.36523049 0.00000000  H -2.19682918 -1.18866400 0.00000000  H -0.09918706 -2.49836280 0.00000000  H -2.22336092 2.37838312 0.00000000  H 2.22336092 -2.37838312 0.00000000 |
| $\mathbf{C}_{\mathbf{6}}\mathbf{H}_{\mathbf{4}}\left( \mathbf{OH} \right)_{\mathbf{2}}$  C 1.20720217 -0.70065789 0.00000000  C 1.20720217 0.68947114 0.00000000  C 0.00509579 1.38532901 0.00000000  C -1.20720217 0.70065789 0.00000000  C -1.20720217 -0.68947114 0.00000000  C -0.00509579 -1.38532901 0.00000000  O 2.42158347 -1.33812988 0.00000000  H 2.15154731 1.21736109 0.00000000  H 0.01538568 2.47005037 0.00000000  O -2.42158347 1.33812988 0.00000000  H -2.15154731 -1.21736109 0.00000000  H -0.01538568 -2.47005037 0.00000000  H -2.28522040 2.29168294 0.00000000  H 2.28522040 -2.29168294 0.00000000 | $\mathbf{C}_{\mathbf{6}}\mathbf{H}_{\mathbf{4}}\left( \mathbf{O} \right)_{\mathbf{2}}$  C 0.00000000 0.00000000 1.43899360  C 0.00000000 1.26587017 0.66802593  C 0.00000000 1.26587017 -0.66802593  C 0.00000000 0.00000000 -1.43899360  C 0.00000000 -1.26587017 -0.66802593  C 0.00000000 -1.26587017 0.66802593  H 0.00000000 2.17696713 1.25374060  H 0.00000000 2.17696713 -1.25374060  H 0.00000000 -2.17696713 -1.25374060  H 0.00000000 -2.17696713 1.25374060  O 0.00000000 0.00000000 -2.65666095  O 0.00000000 0.00000000 2.65666095 |

# References

1 J. C. V. Chagas, B. D. Milanez, V. P. Oliveira, M. Pinheiro Jr, L. F. A. Ferrão, A. J. A. Aquino, H. Lischka and F. B. C. Machado, *J Comput Chem*, 2024, **45**, 863–877.

2 M. Máximo-Canadas, R. S. S. Oliveira, M. A. S. Oliveira and I. Borges, *ACS Omega*, 2025, **10**, 14157–14175.
